# Rapid and Sensitive Multiplex Detection of *Burkholderia pseudomallei*-Specific Antibodies in Melioidosis Patients Based on a Protein Microarray Approach

**DOI:** 10.1371/journal.pntd.0004847

**Published:** 2016-07-18

**Authors:** Christian Kohler, Susanna J. Dunachie, Elke Müller, Anne Kohler, Kemajittra Jenjaroen, Prapit Teparrukkul, Vico Baier, Ralf Ehricht, Ivo Steinmetz

**Affiliations:** 1 Friedrich Loeffler Institut for Medical Microbiology, Greifswald, Germany; 2 Mahidol-Oxford Tropical Medicine Research Unit, Faculty of Tropical Medicine, Mahidol University, Bangkok, Thailand; 3 Centre for Tropical Medicine and Global Health, University of Oxford, Oxford, United Kingdom; 4 Alere Technologies GmbH, Jena, Germany; 5 InfectoGnostics Research Campus, Jena, Germany; 6 Sappasithiprasong Hospital, Ubon Ratchathani, Thailand; 7 Institute of Hygiene, Microbiology and Environmental Medicine, Medical University of Graz, Graz, Austria; University of Tennessee, UNITED STATES

## Abstract

**Background:**

The environmental bacterium *Burkholderia pseudomallei* causes the infectious disease melioidosis with a high case-fatality rate in tropical and subtropical regions. Direct pathogen detection can be difficult, and therefore an indirect serological test which might aid early diagnosis is desirable. However, current tests for antibodies against *B*. *pseudomallei*, including the reference indirect haemagglutination assay (IHA), lack sensitivity, specificity and standardization. Consequently, serological tests currently do not play a role in the diagnosis of melioidosis in endemic areas. Recently, a number of promising diagnostic antigens have been identified, but a standardized, easy-to-perform clinical laboratory test for sensitive multiplex detection of antibodies against *B*. *pseudomallei* is still lacking.

**Methods and Principal Findings:**

In this study, we developed and validated a protein microarray which can be used in a standard 96-well format. Our array contains 20 recombinant and purified *B*. *pseudomallei* proteins, previously identified as serodiagnostic candidates in melioidosis. In total, we analyzed 196 sera and plasmas from melioidosis patients from northeast Thailand and 210 negative controls from melioidosis-endemic and non-endemic regions. Our protein array clearly discriminated between sera from melioidosis patients and controls with a specificity of 97%. Importantly, the array showed a higher sensitivity than did the IHA in melioidosis patients upon admission (cut-off IHA titer ≥1:160: IHA 57.3%, protein array: 86.7%; *p* = 0.0001). Testing of sera from single patients at 0, 12 and 52 weeks post-admission revealed that protein antigens induce either a short- or long-term antibody response.

**Conclusions:**

Our protein array provides a standardized, rapid, easy-to-perform test for the detection of *B*. *pseudomallei*-specific antibody patterns. Thus, this system has the potential to improve the serodiagnosis of melioidosis in clinical settings. Moreover, our high-throughput assay might be useful for the detection of anti-*B*. *pseudomallei* antibodies in epidemiological studies. Further studies are needed to elucidate the clinical and diagnostic significance of the different antibody kinetics observed during melioidosis.

## Author Summary

Melioidosis is a potentially fatal infectious disease caused by the Gram-negative environmental bacterium *Burkholderia pseudomallei*. Since the clinical presentations of melioidosis are extremely variable and no specific signs or symptoms exist, early laboratory-based diagnosis is highly desirable to start appropriate antibiotics. Routine methods for culture detection of *B*. *pseudomallei* are highly specific but take several days for a result, and depending on the clinical sample and other factors, sensitivity can be low. The standard serology test for melioidosis is an indirect hemagglutination assay (IHA) based on crude *B*. *pseudomallei* antigen preparations. Due to the variable prevalence of background seropositivity in endemic areas and the low diagnostic sensitivity of the IHA upon admission, the test is currently not recommended for the diagnosis of melioidosis, but still widely used. Thus, we generated a protein array containing 20 *B*. *pseudomallei* antigens previously shown to have serodiagnostic potential. Our array allows highly specific and sensitive antibody recognition in blood sera and plasmas from patients with melioidosis. The standardized microarray device is simple to use and fast, and is thus applicable in a routine diagnostic laboratory. In this study, the multiplex testing of antibodies in melioidosis sera from different time points after admission allowed the detection of short- and long-term antibodies to various antigens. Further studies will examine the potential role of those antibodies to discriminate different stages of the disease. Furthermore, the protein microarray might be useful in studies aimed at elucidating the exposure of humans and animals to *B*. *pseudomallei* in different parts of the world.

## Introduction

Melioidosis is an often fatal tropical infectious disease caused by the Gram-negative environmental bacterium *Burkholderia pseudomallei* [1, 2]. The disease is known to be highly endemic in Southeast Asia and northern Australia. However, an increasing number of melioidosis case reports or environmental isolation of *B*. *pseudomallei* from other parts of Asia, Africa, the Caribbean, and Central and South America suggest a worldwide, but grossly underreported distribution of *B*. *pseudomallei* between latitudes 20° N and 20° S [3–9]. Recently, Limmathurotsakul and coworkers predicted about 165,000 cases of human melioidosis per year worldwide, from which 89,000 people die [10]. Farmers and indigenous inhabitants of rural tropical areas are population groups at greatest risk of infection, especially in times of heavy rains [1, 2, 5]. Melioidosis usually has an incubation period of 1 to 21 days (mean: 9 days) and causes a wide range of acute or chronic clinical manifestations, including pneumonia, abscesses in various organs, neurological manifestations, or severe septicemia [1, 2, 11–13]. Since *B*. *pseudomallei* is intrinsically resistant to many antibiotics, it requires an immediate diagnosis followed by specific and prolonged antibiotic therapy. Melioidosis has a case fatality rate of around 40% in northeast Thailand [14]. In acute forms, death can occur within 24–48 hours of the onset of symptoms [15, 16].

The rapid diagnosis of melioidosis is still a major obstacle in many potentially endemic parts of the world. Cultural identification of *B*. *pseudomallei* can be difficult, especially in non-endemic areas where clinical suspicion and awareness in the laboratory is low [1, 13, 17]. Even in endemic areas, the culture method has a low sensitivity and might take several days until results are available [18]. In addition, laboratory facilities for microbiological culture are unavailable in many countries of the world where melioidosis is endemic or suspected to be present. Serodiagnostic methods might have the potential to complement direct pathogen detection. The indirect hemagglutination assay (IHA) is the known standard serology test for melioidosis [1, 13, 19, 20]. This assay, based on sheep red blood cells sensitized with crude *B*. *pseudomallei* antigen is simple to perform and inexpensive. However, the diagnostic sensitivity of this approach upon admission is about 56% and a high seropositive background in endemic areas reduces the specificity [21, 22]. The crude preparations are difficult to standardize, and different strains have been used for antigen preparations in different laboratories.

Protein microarrays are an effective approach to perform large scale serological studies and enable a fast, parallel analysis of a multitude of possible antigens [23, 24]. They can be produced and probed in a high-throughput manner and are hence highly standardized [23]. In a previous study, Felgner *et al*. (2009) identified 49 *B*. *pseudomallei* proteinogenic antigens that were significantly more reactive in melioidosis patients than in controls [25]. Based on a selection of 20 of those antigens, we constructed a protein microarray using a robust, commercially available technology that can be used for high-throughput testing in a clinical laboratory [23, 26, 27]. The results of probing 196 melioidosis positive and 210 negative control samples from endemic and non-endemic areas as well as samples from patients with other bacteremia or fungemia demonstrated a high sensitivity and specificity. Moreover, for the first time to the authors’ knowledge, the multiplex detection of short- and long-term antibodies against various protein antigens in melioidosis patients is described.

## Materials and Methods

### Ethics statement

This retrospective study was approved by the ethics committees of Faculty of Tropical Medicine, Mahidol University (Submission number TMEC 12–014); of Sappasithiprasong Hospital, Ubon Ratchathani (reference 018/2555); and the Oxford Tropical Research Ethics Committee (reference 64–11). The study was conducted according to the principles of the Declaration of Helsinki (2008) and the International Conference on Harmonization (ICH) Good Clinical Practice (GCP) guidelines. Written informed consent was obtained for all patients enrolled in the study.

### Bacterial strains and plasmids

The bacterial strains and plasmids used are listed in S1 Table. *Escherichia coli* strains DH5α and expression strain BL21DE3pLysS as well as the *B*. *pseudomallei* strain K96243 were cultured in Luria-Bertani (LB) medium or LB agar at 37°C. Unless stated otherwise, the concentrations of antibiotics added to LB medium for *E*. *coli* were as follows: ampicillin (Ap, Sigma-Aldrich, Germany), 100 μg/ml and/or chloramphenicol (Cm, Sigma-Aldrich, Germany), 25 μg/ml.

### *B*. *pseudomallei* antigen selection, cloning and purification

Twenty *B*. *pseudomallei* antigens with serodiagnostic potential were chosen as targets from studies by Felgner *et al*. (2009) and Suwannasaen *et al*. (2011), and are listed in Table 1. Proteins were selected based on their diagnostic sensitivity and specificity as determined by Felgner *et al*. (2009), their genomic location (Chromosome 1 or 2), their bacterial location (cytoplasm, extracellular, periplasm, membrane/outer membrane), their predicted function (protein folding and stabilization, metabolism, virulence, unknown function etc.) and first of all their solubility in phosphate buffered saline after the freezing and storage process. All protein antigens were analyzed by PSORTb version 3.0.2 (http://www.psort.org/psortb/), and any signal sequences or transmembrane domains were excluded for further cloning. The respective protein encoding DNA fragments were amplified by PCR using specific oligonucleotides (S2 Table) and genomic DNA from *B*. *pseudomallei* K96243 strain as the template. Oligonucleotides were created using primer design software Primer`D`Signer 1.1 (IBA GmbH, Göttingen, Germany). PCR products were digested and cloned using appropriate restriction enzymes and protein expression plasmids (S2 Table). The correctness of all cloned genes was confirmed by DNA sequencing. For protein expression, plasmids were transformed in *E*. *coli* expression strain BL21(DE3)pLysS by heat shock and were grown in LB medium with permanent agitation at 37°C to an optical density (OD_540nm_) of 0.5. Protein expression was induced by adding isopropyl-β-D-thiogalactopyranoside (IPTG, 1 mM final concentration) (Carl Roth GmbH, Germany), and after 3 hours, cells were harvested by centrifugation at 8000 x g and 4°C for 10 minutes. Afterwards, cells were disrupted by six cycles (3 min at 4°C) of ultrasonic homogenizer UP50H (Hielscher Ultrasonics GmbH, Germany), and the lysates were centrifuged at 4°C and 12000 x g for 30 minutes. Supernatants were stored at -20°C until use. The protein purification of *Strep*-tag or *His*-tag recombinant proteins was performed by using *Gravity flow Strep Tactin-Sepharose Columns* (IBA GmbH, Göttingen, Germany) or *Ni-NTH Agarose* (Qiagen, Germany) according to the manufacturers’ instructions. Afterwards, purified proteins were dialyzed against Dulbecco’s Phosphate Buffered Saline (DPBS) (Gibco-life technologies, USA), and their purity was confirmed by SDS page (S1 Fig). Recombinant proteins were stored at -20°C until use for protein array construction.

**Table 1:** Characteristics of the *Burkholderia pseudomallei* antigens used in this study.

	Locus Bps^a^	Used name for protein array	Protein name^a^	Definition^a^	Function^a^	Genome location^b^	Expressed amino acids^c^	Tag^d^	Protein location^e^
**1**	BPSL0280	BPSL0280	FlgK	flagellar hook-associated protein FlgK	cell motility	Chr. 1	total (aa 667)	Strep	e
**2**	BPSL1445	BPSL1445	-	putative lipoprotein	unknown	Chr. 1	aa 23 to 195	Strep	e/m
**3**	BPSL1661	BPSL1661-1001	-	putative hemolysin-related protein	unknown	Chr. 1	aa 1 to 683	Strep	e
**4**	BPSL1661	BPSL1661-1002	-	putative hemolysin-related protein	unknown	Chr. 1	aa 1001 to 2000	Strep	e
**5**	BPSL2030	BPSl2030	-	putative exported protein	unknown	Chr. 1	aa 23 to 186	Strep	e
**6**	BPSL2096	BPSL2096	-	hydroperoxide reductase	detoxification	Chr. 1	total (aa 182)	Strep/His	c
**7**	BPSL2520	BPSL2520	-	putative exported protein	unknown	Chr. 1	aa 22 to 198	Strep	e
**8**	BPSL2522	BPSL2522	-	outer membrane protein a precursor	unknown	Chr. 1	aa 23 to 224	Strep	om
**9**	BPSL2697	BPSL2697	GroEL	60 kDa molecular chaperone GroEL	protein folding and stabilisation	Chr. 1	total (aa 546)	Strep/His	c
**10**	BPSL2698	BPSL2698	GroES	10 kDa molecular chaperone GroES	protein folding and stabilisation	Chr. 1	total (aa 97)	Strep	c
**11**	BPSL3319	BPSL3319	FliC	flagellin	cell motility	Chr. 1	total (aa 388)	Strep	e
**12**	BPSS0476	BPSS0476	GroES	10 kDa chaperonin	protein folding and stabilisation	Chr. 2	total (aa 96)	Strep	c
**13**	BPSS0477	BPSS0477	GroEL	60 kDa chaperonin	protein folding and stabilisation	Chr. 2	total (aa 546)	Strep/His	c
**14**	BPSS0530	BPSS0530	-	conserved hypothetical protein	protein secretion, Type VI system	Chr. 2	total (aa 453)	Strep	c
**15**	BPSS1385	BPSS1385	-	ATP/GTP binding protein	unknown	Chr. 2	total (aa 328)	Strep	c
**16**	BPSS1516	BPSS1516	BopC	effector protein[28]	virulence, effector protein	Chr. 2	total (aa 469)	Strep	c
**17**	BPSS1525	BPSS1525-79	BopE	G-nucleotide exchange factor	virulence, effector protein	Chr. 2	aa 79 to 261	Strep	e
**18**	BPSS1532	BPSS1532-344	BipB	putative cell invasion protein	virulence, effector protein	Chr. 2	aa 1 to 344	Strep/His	e
**19**	BPSS1722	BPSS1722	Mdh	malate dehydrogenase	citrate cycle	Chr. 2	total (aa 327)	Strep	c
**20**	BPSS2141	BPSS2141	OppA	periplasmic oligopeptide-binding protein precursor	transport	Chr. 2	aa 40 to 554	Strep	p

^a^ Locus name, protein name, definition and function were used from *B*. *pseudomallei* strain K96243 and obtained from Kyoto Encyclopedia of Genes and Genomes (KEGG) (www.genome.jp/kegg/)

^b^ Location of the respective gene in the genome of *B*. *pseudomallei* K96243. Chr. 1 = chromosome 1; Chr.2 = chromosome 2

^c^ Shows length and/or expressed part of the respective protein. Proteins were expressed without signal sequences and membrane domains. aa—amino acid; total—whole protein

^d^ Tag indicates the used C-terminal amino acid sequence used for purification. Strep–*Strep*-tag; His–His-tag

^e^ Protein location was determined by PSORTb 3.0.2 (Prediction of Protein Sorting Signals and Location Sites in Amino Acid Sequences) http://www.psort.org/psortb/

c–cytoplasm, e–extracellular, m–membrane, om–outer membrane, p—periplasm

### Blood sera and plasma samples

Patients included in the study formed a consecutive series. Sera and plasma from culture-confirmed melioidosis patients were collected from September 2012 to November 2014 in the highly endemic area of Ubon Ratchathani, Thailand. (Table 2) as described previously [29]. Negative control sera (n = 100) consisted of 50 sera of healthy individuals from Ubon Ratchathani (endemic), 25 sera from healthy individuals with diabetes from the same region, and 25 sera from healthy individuals in Bangkok. Further negative controls were drawn from healthy individuals and patients with other bacteremia or fungemia in the non-endemic area of Greifswald (Germany). Sera from melioidosis patients were taken within the first week (´week 0´, n = 75) post-admission (*p*.*a*.), 12 weeks *p*.*a*. (´week 12´, n = 50) and 52 weeks *p*.*a*. (´week 52´, n = 46). Endemic samples (week 0, 12 and 52) were considered melioidosis positive if *B*. *pseudomallei* was isolated from blood, pus, or any other body fluid. The majority of patients were male (week 0, 12 or 52: 68%, 72% and 71.7%, respectively) with a median age of 55 years. IHA titers were performed on all sera drawn in Thailand as described previously [30, 31] (Table 2). A serum was classified as positive if the cut-off for the IHA titer was equal to or higher than 160. This cut off has been widely used in studies in Thailand [32, 33], although lower cut offs were used in other endemic regions [22, 34], possibly to methodological variations and/or less background seropositivity. The IHA titers of the analyzed plasma samples were not determined. The melioidosis negative sera (n = 85) drawn in the non-endemic region of Germany consisted of sera from patients with other bacteremia or fungemia (n = 60) and healthy blood donors (n = 25) (Table 2). IHA titers of these sera were also not determined. All sera or plasmas were stored at -80°C.

**Table 2:** Characteristics of the sera and plasmas used in this study.

Sera / Plasmas	Number	Median IHA	Mean / Median Age	Sex^b^	Diabetes	Blood Culture	Mean / Median ADM Samples^#^
sera melioidosis positive, week 0: total	75	160	55 / 56	24 F / 51 M	51 pos / 24 neg	51 pos / 24 neg	5 / 5
sera melioidosis positive, week 0: survivors	40	320	53 / 54.5	10 F / 30 M	30 pos / 10 neg	20 pos / 20 neg	6 / 5
sera melioidosis positive, week 0: non-survivors	35	80	57 / 57	14 F /21 M	21 pos / 14 neg	31 pos / 4 neg	5 / 5
sera melioidosis positive, week 12	50	320	54 / 54	14 F / 36 M	35 pos / 15 neg	24 pos /26 neg	5 / 5
sera melioidosis positive, week 52	46	80	53.5 / 54	13 F / 33 M	32 pos / 14 neg	22 pos / 24 neg	5 / 5
sera healthy, endemic (Ubon Ratchathani) and non endemic (Bangkok, Thailand)	100	10	46 / 44	47 F / 53 M	74 pos / 26 neg	-	-
sera healthy, non-endemic (Greifswald, Germany)	25	n.d.	37 / 33	12 F / 13 M	n.d.	-	-
sera bacteremia/fungemia, non-endemic (Greifswald, Germany)	60	n.d.	59 / 62	22 F / 38 M	n.d.	60 pos / 0 neg	9.4 / 5.5^a^
plasmas melioidosis negative	25	n.d.	52 / 51	8 F / 17 M	0 pos / 25 neg	-	-
plasmas melioidosis positive: total	25	n.d.	54 / 54	8 F / 17 M	18 pos / 7 neg	15 pos / 10 neg	7 / 6
plasmas melioidosis positive: survivor	15	n.d.	52 / 54	4 F / 11 M	11 pos / 4 neg	5 pos / 10 neg	7 / 8
plasmas melioidosis positive: non-survivor	9	n.d.	57 / 62	4 F / 5 M	6 pos / 3 neg	9 pos / 0 neg	6 / 6
plasmas melioidosis positive: unknown outcome	1	n.d.	52	M	pos	pos	6

n.d.–not determined

^#^ ADM—Number of days from date of hospital admission to date of serum/plasma sample draw.

^a^ Number of days from date of positive blood culture to date of serum sample draw.

^b^ F–female, M—male

### *Burkholderia pseudomallei* protein array construction and preparation

All purified proteins were spotted on a 4.2 x 4.2-mm glass microarray surface with a spotted area of 3.6 x 3.6 mm and incorporated in the *ArrayStrip* system provided by Alere Technologies GmbH (Germany), resulting in the first-generation *B*.*pseudom*.*01-Array*. Recombinant proteins were covalently immobilized as triplicates at five different concentrations (0.01 to 0.45 mg/ml); subsequently bovine serum albumin was immobilized to a concentration of 0.5 mg/ml on the array. Horseradish peroxidase (HRP) and purified IgG and IgM antibodies from different species (humans, mice, pigs, sheep, goats and cattle) served as positive controls, and spotted bovine serum albumin (BSA) functioned as the negative control. After manufacturing, each single *ArrayStrip* was sealed under a noble gas (argon) atmosphere into nontransparent bags and stored at 4°C until use.

### Protocol testing for IgG from human sera and plasmas

Antibody detection using the *B*.*pseudom*.*01-Array* was performed according to a previously optimized manufacturer’s protocol. Briefly, protein arrays were first incubated with washing buffer (1xPBS/0.05% Tween 20/0.25% TritonX100) at 37°C and 400 rpm for 5 minutes. Afterwards, protein arrays were incubated with blocking buffer (1xPBS/0.05% Tween 20/0.25% TritonX100 and 2% Blocking Reagent (No 11 096 176 001; Roche, Switzerland)) at 37°C and 300 rpm for 5 min in order to block unspecific binding sides. Subsequently, diluted sera and plasmas (10^−3^) were incubated for 30 min at 37°C and 300 rpm. After a washing step as described above (37°C, 400 rpm, 5 min), the protein arrays were incubated with a diluted (10^−3^) HRP coupled anti-human IgG antibody (Sigma-Aldrich, USA) at 37°C and 300 rpm min for 30 min. To avoid strong background signals, protein arrays were washed again twice with washing buffer (37°C, 400 rpm, 4 min) and finally incubated with the specific substrate D1 (Alere Technologies GmbH, Jena, Germany) for exactly 10 min without shaking at 25°C. Finally, the protein arrays were read out by the *ArrayMate* and data were analyzed using *IconoClust* software according to the manufacturer’s specifications (both by Alere Technologies GmbH, Germany). The following parameters for evaluating the arrays were used:

The normalized intensities (NI) of the spots were determined as NI = 1-(*M*/BG), where *M* is the average intensity of the spot and BG is the intensity of the local background. Hence, results range between 0 (no signal) and 1 (maximal intensity). Spot intensities of at least 0.3 were defined as a specific antibody response to the respective antigens. The recognition of at least two different antigens per serum or plasma with signal intensities above 0.3 was considered melioidosis positive. Sensitivities and specificities of the IHA and the protein array were calculated using following equations: sensitivity = ∑ true melioidosis positive tested individuals / ∑ total melioidosis positive individuals; specificity = ∑ true melioidosis negative tested individuals / ∑ total melioidosis negative individuals. Readers were blind to clinical outcome and to results of other tests at the time of reading.

### Statistical analyses and data visualization

The two-sided Fisher's exact test was used to show whether the proportions of positive and negative signals differ between individual groups, i.e, melioidosis positive and negative samples. [35]. Fisher's exact test was carried out for the signals of each spotted substance in the microarray, using R as the language for statistical computing (R Core Team, 2015. R: A language and environment for statistical computing. R Foundation for Statistical Computing, Vienna, Austria. URL http://www.R-project.org/). P < 0.01 was considered statistically significant. In this study, the software programs *GraphPadPrism* 5.0 (GraphPad software, Inc., USA), *Excel* 2010 (Microsoft Corporation, USA) and *Multi experiment Viewer* 4.9.0 (TM4 suite, USA) were used for visualization of the data.

## Results

### Antigen selection and protein microarray construction

**Fig 1:**
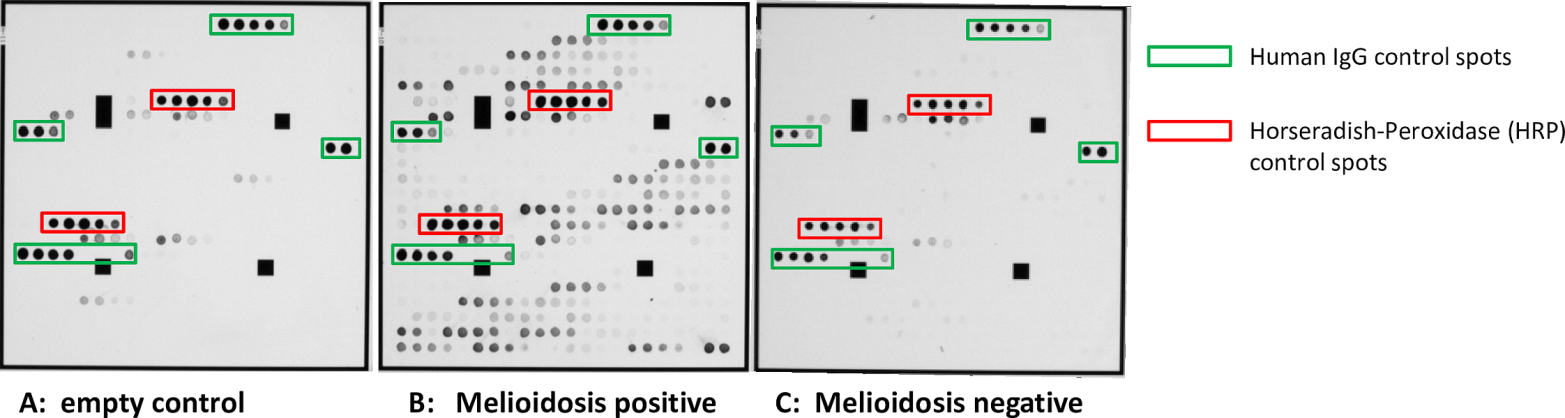
Construction of a *B*. *pseudomallei* protein microarray. The protein array was spotted with 20 different *B*. *pseudomallei* proteins and further internal positive and negative controls, for a total of 445 protein spots. All proteins were applied in triplicate and at five dilutions (0.01 mg/ml to 0.45 mg/ml) on glass slides, including human IgG and IgM controls and further other internal controls, i.e., murine IgG and IgM, bovine IgG, porcine IgG, caprine IgG and ovine IgG controls. All protein arrays were incubated **A** only with anti-human-IgG antibodies (empty control), **B** with melioidosis-positive or **C** melioidosis-negative blood sera or blood plasmas at 1:1000 dilutions (**A, B** and **C** are representative images). IgG antibodies bound to *B*. *pseudomallei* antigens were detected using horseradish-peroxidase (HRP) linked secondary anti-human-IgG antibodies and 3,3’,5,5’-tetramethyl-benzidine (TMB), which caused a blue precipitate. Protein arrays were read out by the ArrayMate (Alere Technologies GmbH, Germany). Highlighted are human IgG controls (green rectangle) and horseradish-peroxidase controls (red rectangle); all other controls are not shown.

In this study, a protein microarray was developed containing 20 *B*. *pseudomallei* proteins, previously identified by Felgner *et al*. (2009) to have serodiagnostic potential in melioidosis [25]. All proteins were expressed in *E*. *coli*, purified (S1 Fig), and spotted at five increasing concentrations (0.01 to 0.45 mg/ml) on the glass microarray surface (Fig 1). Among those antigens are cytoplasmic proteins (n = 9), extracellular proteins (n = 9), outer membrane/membrane proteins (n = 2), and periplasmic proteins (n = 1) (Table 1). The antigens are predicted to be involved in protein folding and stabilization, cell motility, detoxification, virulence, and transport, and may have yet unknown functions. In contrast to the protein microarray platform used by Felgner *et al*., recombinant proteins were exempted from signal sequences or transmembrane domains to maintain them in a soluble state. The whole protocol starting from sera or plasma incubation to final data analysis takes about two hours (S2 Fig) [23, 26, 27]. The protocol uses pure and highly standardized chemicals and antibodies that are available worldwide (see Material and Methods).

### Detection of anti-*B*. *pseudomallei* IgG antibodies in sera and plasmas of melioidosis patients

**Fig 2:**
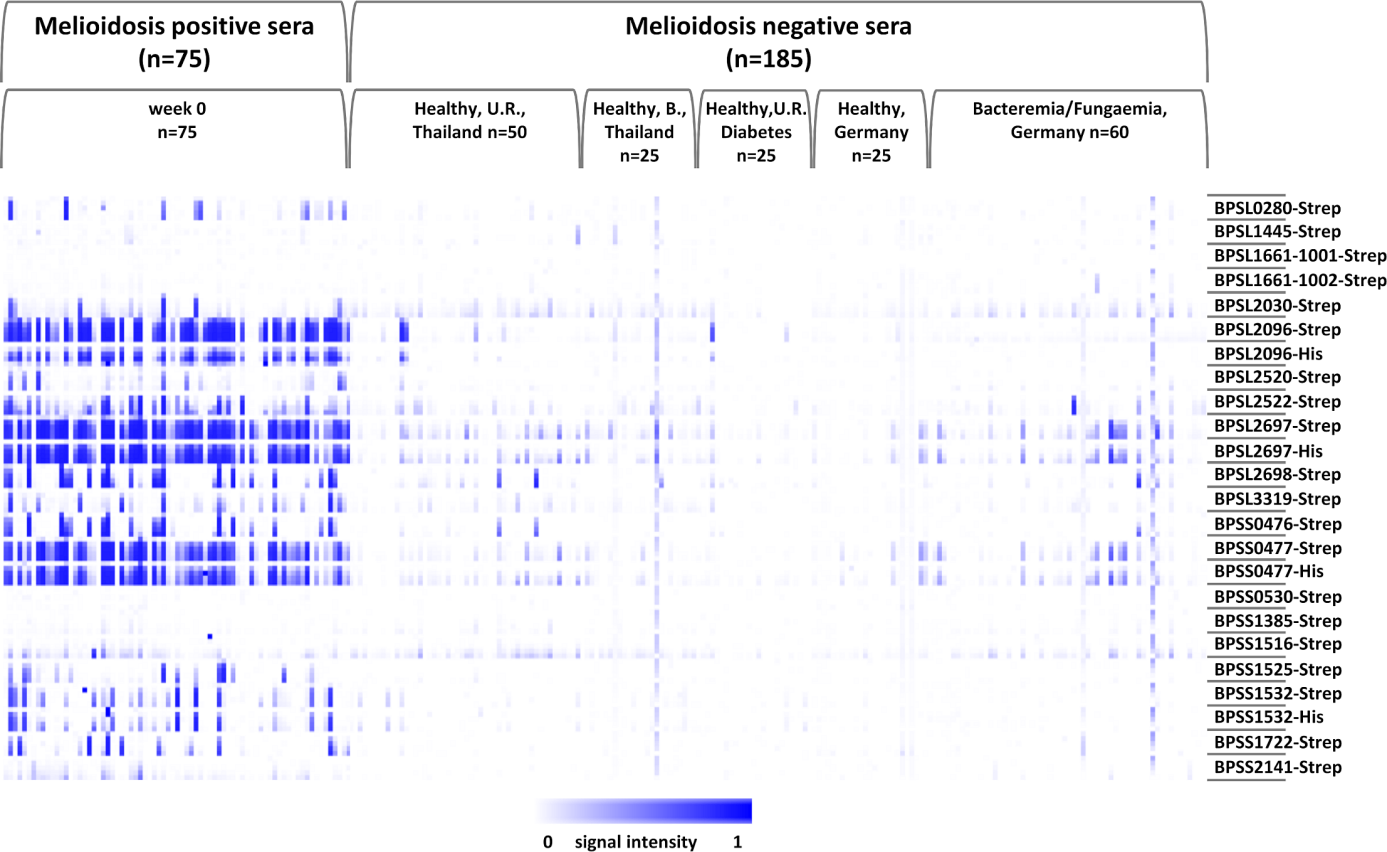
Heatmap of probing a collection of melioidosis-positive sera and negative control sera. Protein arrays containing 20 *B*. *pseudomallei* recombinant proteins were probed with 260 melioidosis and nonmelioidosis sera. The melioidosis positive sera (n = 75) were drawn at week 0 (*p*.*a*.) from patients with *B*. *pseudomallei* infections. All positive sera were sampled in Ubon Ratchathani, Thailand. Negative control sera of healthy persons (n = 125) were sampled in the endemic regions of Thailand ((Ubon Ratchathani (U.R.) and Bangkok (B.)), Thailand and non-endemic region of Greifswald, Germany. Additionally, further negative control sera of patients with other bacteremia or fungaemia (n = 60) were used from the non-endemic region of Greifswald. Not shown are the results of incubations with meliodosis-positive sera obtained 12 and 52 weeks after admission. The antigens are shown in rows with five increasing concentrations per protein, and the patient samples are represented in columns. Array signals are reflected by the intensities of the color (white to blue) inside the boxes. The heatmap was created using Multi experiment Viewer (MeV 4.9.0) from TM4 suite, USA.

**Fig 3:**
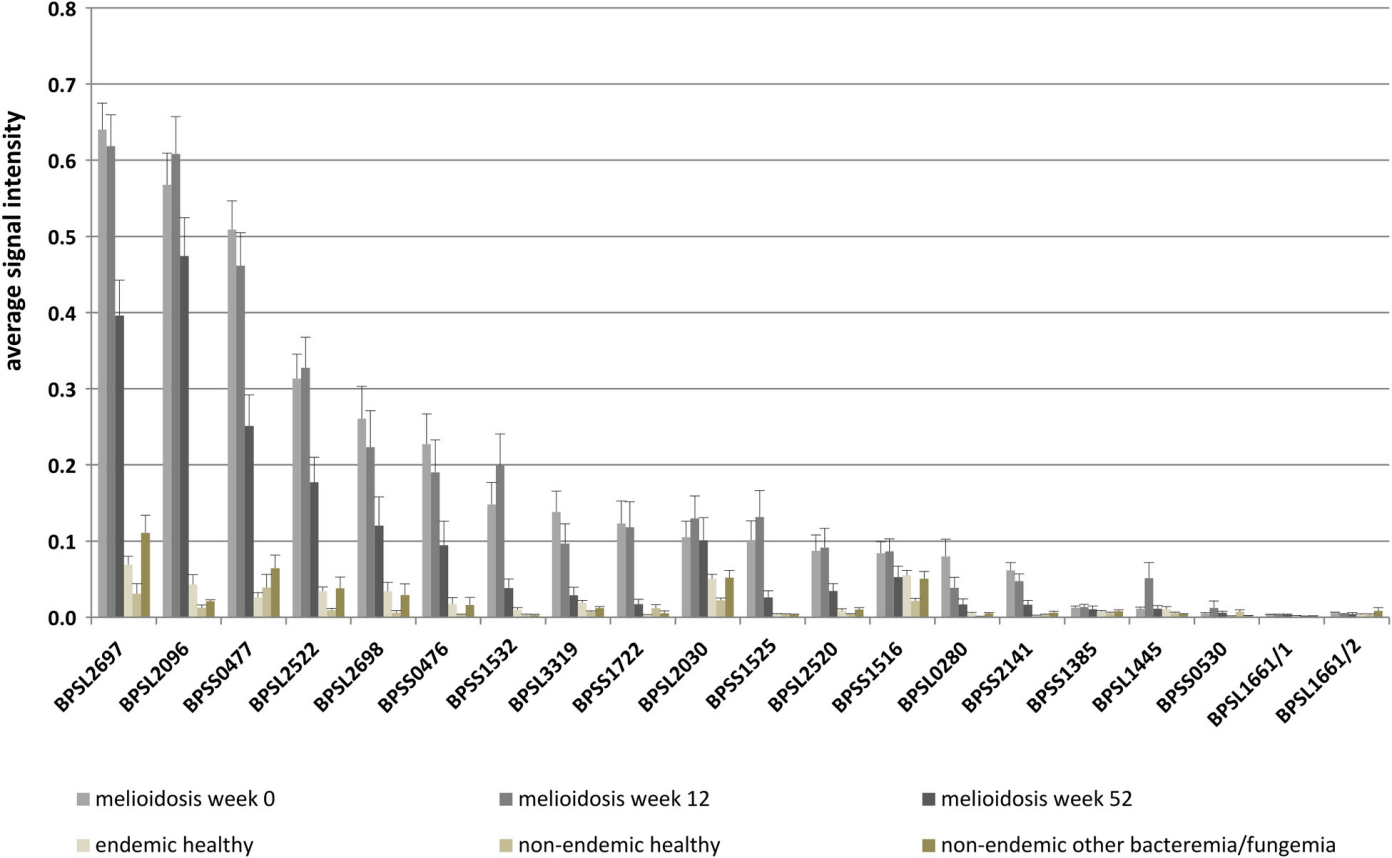
Average signal intensities of IgG antibodies bound to *B*. *pseudomallei* proteins probed with melioidosis-positive and negative control samples. The diagram shows the average signal intensity of each antigen (spotted protein solution of 0.45 mg/ml) incubated with sera from melioidosis-positive groups (week 0, 12 and 52 *p*.*a*.), healthy control individuals from endemic and non-endemic areas, as well as samples from patients with other bacteremia or fungaemia obtained in the non-endemic area of Greifswald. Not shown are values for antigens with His-tag. Error bars indicate standard error of the mean (SEM).

Our protein array was validated using different groups of sera of melioidosis patients (n = 171) or negative control individuals (n = 185) (Table 2). In total, three melioidosis positive groups (week 0, 12 and 52 *p*.*a*.) and two melioidosis negative groups (healthy individuals from Thailand/Germany and patients with other types of bacteremia/fungaemia from Germany) were used for a parallel and comprehensive analysis of human IgG reactivity. The positive sera of patients upon admission (week 0) were clearly distinguishable from all negative control sera (endemic and non-endemic regions) and from sera of patients with other types of bacteremia or fungaemia (Fig 2). Furthermore, all melioidosis-positive sera taken at weeks 12 and 52 *p*.*a*. were also found to be highly distinguishable from all negative control groups (S3 Fig). We observed strong signal intensities even for the low antigen concentrations (0.01 or 0.05 mg/ml antigen), and most antigens showed signal intensities greater than 0.3 at a concentration of 0.45 mg/ml, with a median of 4 recognized antigens at this antigen concentration (S4 Fig). Therefore, we used this concentration for all further analyses. In total, 17 of 20 antigens showed signal intensities above 0.3. The strongest average signal intensities were found for antigens BPSL2697 and BPSL2096, followed by BPSS0477, BPSL2522, BPSL2698, BPSS0476 and BPSS1532 (Fig 3). Lower signal intensities were found for BPSL3319, BPSS1722, BPSL2030, BPSS1525, BPSL2520, BPSS1516, BPSL0280, BPSS2141, BPSL1445 and BPSS0530 (Fig 3). Importantly, no signals could be measured for antigens BPSS1385, BPSL1661-1001 and -1002, although these proteins have been previously described as serodiagnostic marker proteins [25]. No influence could be observed for the nature of protein tags (*His*- or *Strep*-tag) for BPSL2697, BPSL2096, BPSS0477, BPSS1532 (S2 File). Hence, the results for antigens purified with *His*-tag are not further discussed.

In order to elucidate the discriminatory power of the single antigens further, all different groups of melioidosis-positive sera (S5 Fig) were compared with the various controls using the two-sided Fisher's exact test as described by Glantz [35]. Comparisons of sera from healthy donors from both endemic and non-endemic regions with that of melioidosis-positive sera of week 0 *p*.*a*. showed thirteen significantly recognized antigens, twelve antigens from sera of week 12 *p*.*a*. and six antigens from week 52 *p*.*a*. (comparisons 01, 02 and 03 shown in S1 File). Comparisons using sera of patients with other bacteremias or fungaemias revealed eleven significantly recognized antigens from melioidosis-positive sera of week 0 and 12 and four antigens from sera of week 52 (comparisons 05, 06 and 07 shown in S1 File). Testing a higher number of sera will likely increase the number of significantly recognized *B*. *pseudomallei* antigens. Thirteen of the serodiagnostic marker proteins found by Felgner *et al*. (2009) were confirmed here.

Since plasma is routinely drawn in clinical practice, we additionally examined blood plasmas (week 0 *p*.*a*.) of melioidosis-positive (n = 25) and -negative (n = 25) individuals. As shown for sera, melioidosis-positive plasmas were also highly distinguishable from negative control plasmas (S6 Fig and S7 Fig). Compared to the respective sera, almost identical numbers of antigens per plasma were recognized (S8 Fig). In addition, no significant differences in signal intensities per antigen could be observed when sera and plasma samples were used from the same patient (S9 Fig). In one melioidosis plasma sample, we found positive signals for BPSL1661-1002, which were not observed for any positive serum sample. Unfortunately, the corresponding serum sample was not available. However, as shown for sera, a total of 17 of 20 *B*. *pseudomallei* antigens were recognized by at least one melioidosis-positive plasma sample. Our results indicate that in addition to blood sera, also blood plasmas can be used to detect antibodies against *B*. *pseudomallei* in our protein microarray system.

### Short- and long-term antibody responses to different antigens

**Fig 4:**
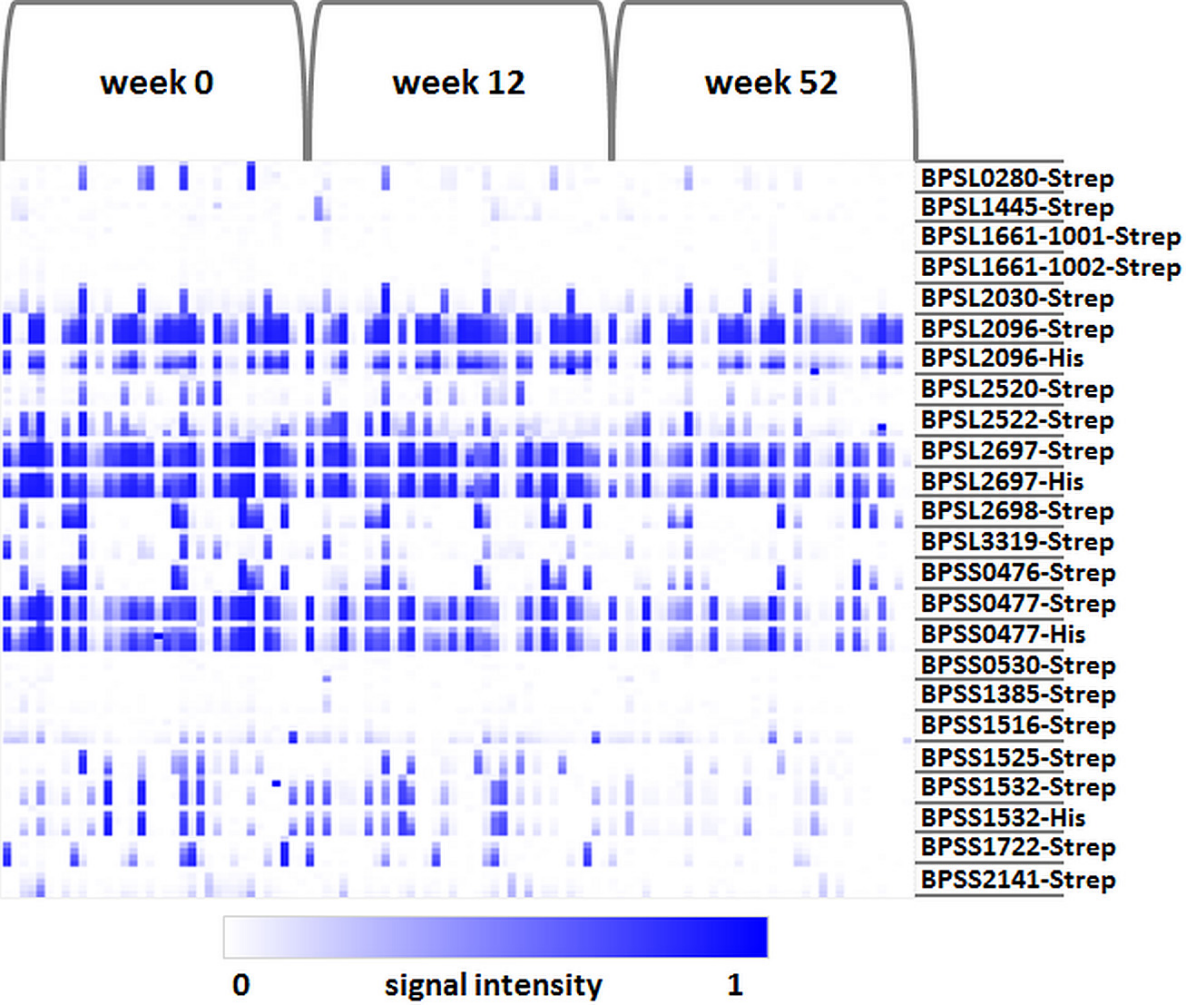
Experimental timeline of probing a collection of positive sera drawn from single melioidosis patients (n = 36) upon admission (week 0) and after 12 and 52 weeks *p*.*a*. All sera were sampled in Ubon Ratchathani, Thailand. The antigens are shown in rows with five increasing concentrations per protein, and the patient samples are represented in columns. Array signals are reflected by the intensities of the color (white to blue) inside the boxes. The heatmap was created using Multi experiment Viewer (MeV 4.9.0) from TM4 suite, USA.

**Fig 5:**
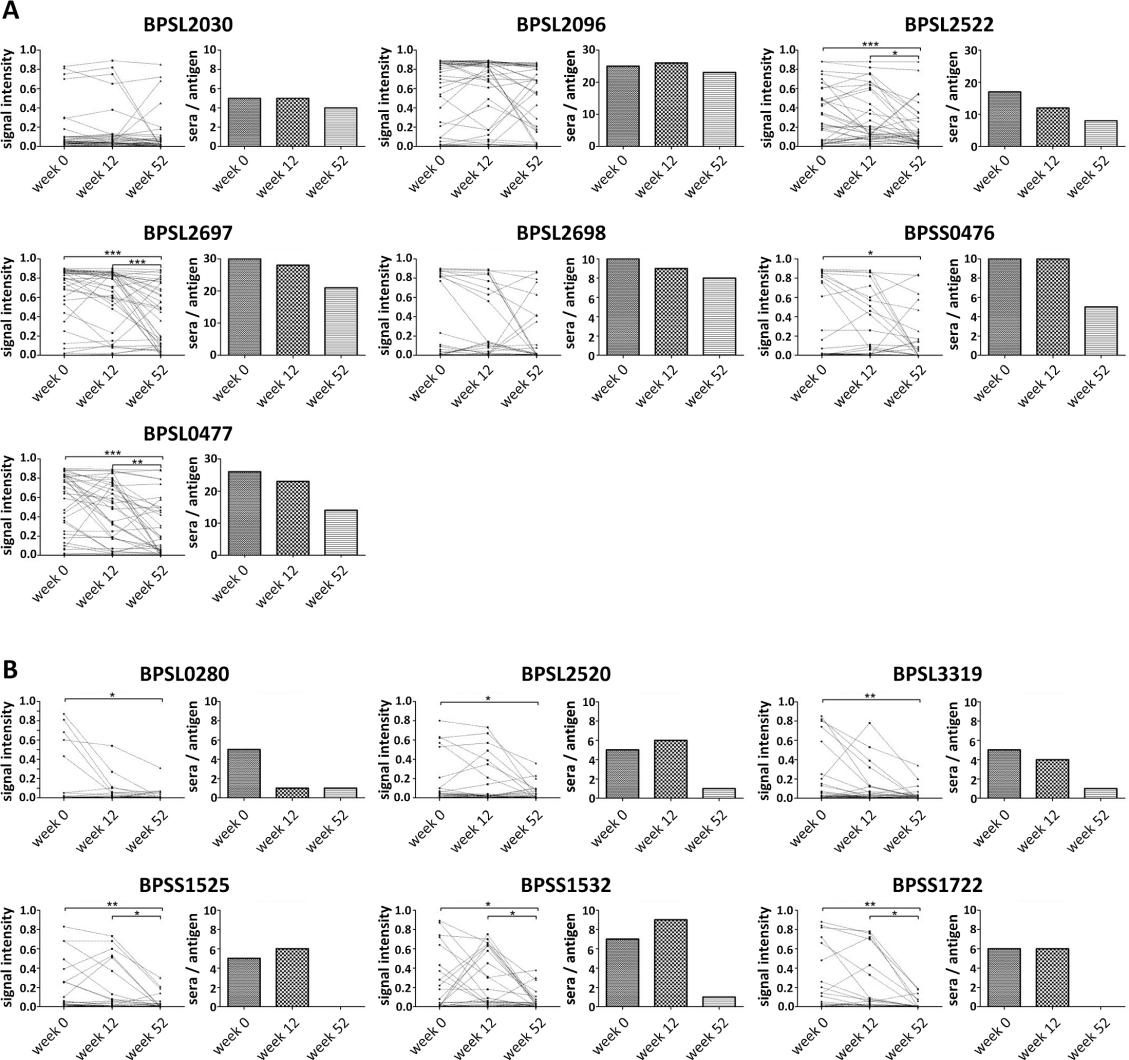
Development of signal intensities of grouped antigens inducing a long-term antibody response (A) and a short-term antibody response (B). Sera of individual patients (n = 36) were drawn upon admission (week 0 *p*.*a*.), 12 and 52 weeks *p*.*a*. Two graphs per antigen are shown. Left: the mean signal intensity per serum. Right: number of sera recognizing the respective antigen. Figures include only data of antigens found to be significantly recognized by melioidosis-positive sera. Statistical analyses were performed using repeated-measures ANOVA followed by Bonferroni's Multiple Comparison test comparing signal intensities measured in sera of week 0, 12 and 52 *p*.*a*. (*p<0.05; **p<0.01; ***p<0.001)

Depending on many different parameters, antigens can elicit antibody responses of different durations. Here, we used melioidosis-positive sera drawn at weeks 0, 12 and 52 *p*.*a*. from individual patients (n = 36) to investigate the antibody responses to the different protein antigens over a prolonged period of time. In general, signal intensities of almost all antigens and the number of antigens detected declined over time (Figs 3 and 4). Two groups of differentially recognized antigens could be described. Antigens of the first group (BPSL2030, BPSL2096, BPSL2522, BPSL2697, BPSL2698, BPSS0476 and BPSS0477) induced a relatively strong, constant antibody response over a prolonged period of time. Even 52 weeks after patient admission, an antibody response against these antigens could be detected in at least 50% of sera (Fig 5A). Recognition of those antigens at weeks 12 and 52 *p*.*a*. was observed in sera which were positive for these antibodies at week 0 *p*.*a*. but also in sera which were negative for those antibodies at week 0 *p*.*a*. (Fig 5A). In contrast, antigens of group 2 (BPSS1532, BPSL3319, BPSS1722, BPSL2030, BPSS1525, BPSL2520) did not show significant recognition in sera from 52 weeks *p*.*a*. (Fig 5B). Interestingly, three antigens of group 1 (BPSL2030, BPSL2096 and BPSS0476) showed the same or a higher number of significant signals (signal intensity ≥ 0.3) if incubated with sera of week 12 *p*.*a*. compared to sera of week 0 (Fig 5A). The same was observed for four antigens (BPSL2520, BPSS1525, BPSS1532 and BPSS1722) of group two antigens (Fig 5B). Among group two members, particularly the antigen BPSL0280 induced only a very short antibody response. After only 12 weeks *p*.*a*., the average signal intensity and number of significant signals was similar to the signals observed for sera obtained at 52 weeks *p*.*a*. (Fig 5B). Importantly, the categorization of antigens into the two groups was confirmed for the complete set of melioidosis sera, including patients where only sera from single time points were available (Table 2 and S3 File).

By using a multiplex detection approach, we revealed for the first time that different *B*. *pseudomallei* protein antigens induce long- and short-term antibody responses. Thus, the identified groups of antigens might have the potential to distinguish between more recent *B*. *pseudomallei* infections and infections which occurred further in the past.

### Sensitivity and specificity

**Fig 6:**
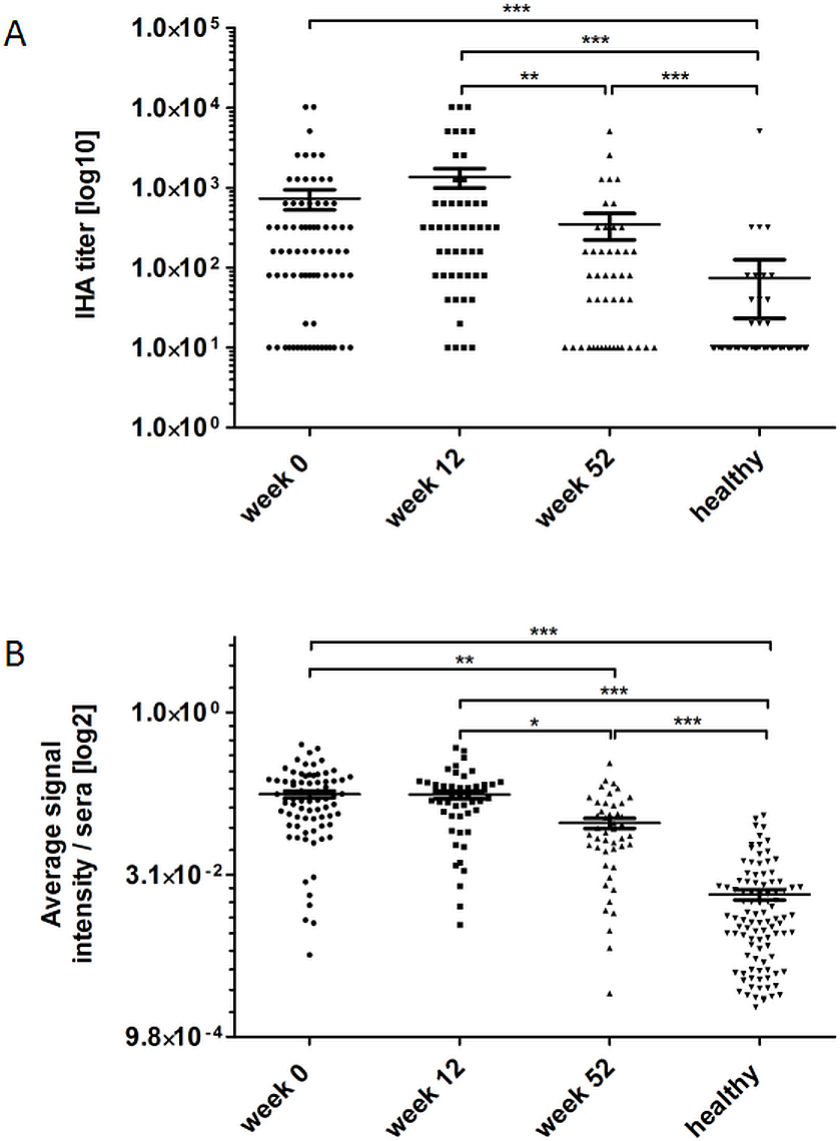
Measured IHA titers (A) and protein-array–derived average signal intensities (B) per serum. Melioidosis-positive and -negative samples were drawn in endemic areas of Thailand. Additionally, means with standard error of the mean (SEM) are shown for each group. The IHA titer was determined using the indirect hemagglutionation assay described elsewhere (http://www.melioidosis.info/home.aspx). Statistical analyses were performed using the Kruskal-Wallis test followed by Dunn's Multiple Comparison test, comparing titers or signal intensities measured in sera of weeks 0 (n = 75), 12 (n = 50), and 52 (n = 46), as well as from healthy individuals (n = 100) (*p<0.05; **p<0.01; ***p<0.001).

**Fig 7:**
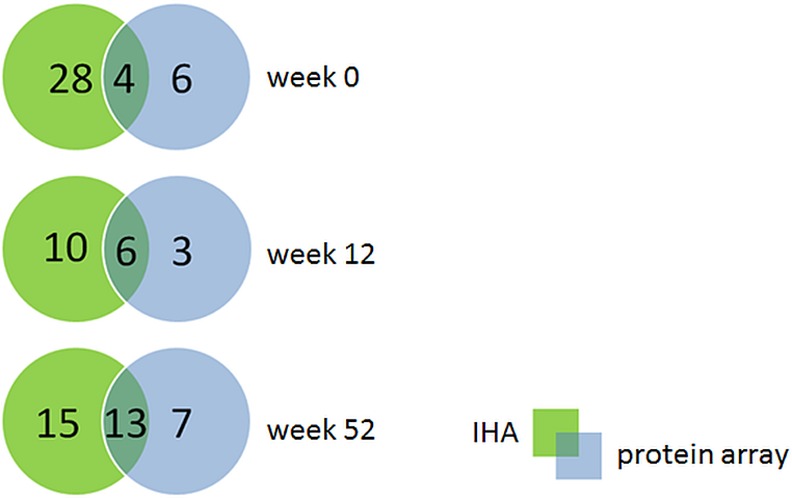
Comparison of false-negative signals between IHA and the protein array. VENN diagrams show the false-negative signals of IHA and the protein array using the melioidosis-positive sera of weeks 0 (n = 75), 12 (n = 50), and 52 (n = 46). Green shows the false-negative signals only for the IHA and blue only for the protein array. The overlaps are false-negative signals obtained by both methods. The cut-off IHA titer used was ≥160.

We further compared sensitivity and specificity of IHA titer values with those results obtained from protein array experiments. In general, mean and median IHA titer measured in sera from patients at admission week 0 *p*.*a*. were higher compared to IHA titer in sera of week 52 *p*.*a*. and negative control sera from Thailand, whereas sera of week 12 *p*.*a*. showed the highest IHA titer measured (Table 2 and Fig 6A). Average signal intensities obtained from protein array experiments showed the same tendencies, but only sera of week 0 *p*.*a*. correlated with the IHA titer values (week 0: r_sp_ = 0.3470, *p* = 0.023; week 12: r_sp_ = 0.2743, *p* = 0.054; week 52: r_sp_ = 0.1602, *p* = 0.2877; healthy: r_sp_ = 0.1107, *p* = 0.2728) (Fig 6B). However, from 75 tested sera of patients upon admission (week 0 *p*.*a*.), 32 sera had an IHA titer lower than 160 and were classified as melioidosis negative. When these 75 sera were analyzed using the protein arrays, only 10 sera gave less than two significant signals to two different antigens and had to be classified as melioidosis negative. Six sera were solely classified as melioidosis negative by the protein array, as opposed to 28 sera classified as negative by IHA. Four sera were classified as melioidosis negative by both methods (Fig 7). Thus, the sensitivity of the protein array (86.7%) was clearly higher than that of the IHA (57.3%), as shown in Table 3. No significant differences in sensitivities could be observed using sera drawn at 12 or 52 weeks *p*.*a*. (Table 3). The specificities of the IHA test and the protein array were barely distinguishable, with 96% and 97%, respectively.

**Table 3:** Comparative calculation of IHA and protein array sensitivity.^#^

	week 0		week 12		week 52	
Cut-off 1:160	IHA	protein array	IHA	protein array	IHA	protein array
positive	43	65	34	41	18	26
negative	32	10	16	9	28	20
sensitivity	**57.3%**	**86.7%**	68%	82%	39.1%	56.5%
*p* value	0.0001		0.1652		0.1436	

^#^ Table shows the sensitivity of both techniques. Cut off for IHA titer was greater or equal 160. Shown are values for week 0, week 12 and week 52 *p*.*a*. Statistical analysis was performed using the Fisher's exact test. *p* values smaller than or equal 0.01 were assumed to differs significantly between both techniques.

## Discussion

The alarmingly large number of predicted melioidosis cases worldwide with a high mortality [10] emphasizes the need to improve the current diagnostic tools to detect *B*. *pseudomallei* infections. The methods for the analysis of immune responses during infection have recently been expanded by including protein microarrays that target pathogen-specific antigens [36–38]. Protein microarrays have the potential advantage of overcoming the limitations of a more or less monoplex antibody detection when single antigens are used [20, 39]. The application of single antigens and thus the restriction to certain epitopes might limit the sensitivity and specificity of diagnostic serological assays. Recently, a protein array approach was used as an antigen discovery platform, and a significant number of serodiagnostic marker proteins of *B*. *pseudomallei* were identified that were more reactive in melioidosis patients compared to controls [25, 40].

Based on the work by Felgner *et al*. [25], we selected 20 antigens to develop a *B*. *pseudomallei* protein microarray using a miniaturized technical platform, which can be automated and is applicable in a routine setting. The validation of our microarray with sera taken from patients at defined time points after admission demonstrates a significantly higher sensitivity of our protein array to detect melioidosis upon admission compared to the standard IHA (86.5% vs 57.3%, respectively). In our study, 13 of the 20 proteins identified by Felgner *et al*. were confirmed as specific serodiagnostic markers. Seven proteins were not statistically significantly recognized by melioidosis-positive sera compared to control sera. Two proteins were not recognized by any serum or plasma tested. This discrepancy to the results found by Felgner *et al*. (2009) may be explained by the following factors: i. All proteins used in this study were free of any transmembrane domains and/or any signal sequences. ii. In contrast to the *in vitro* translation system used by Felgner *et al*., proteins in our study were expressed in *E*. *coli* and purified. iii. Our miniaturized array system used very different protocols for sera incubation, washing steps, and the detection system. In summary, all these factors might have led to the discrepant recognition of diagnostic proteins.

Based on ten of their identified serodiagnostic proteins, Felgner *et al*. developed an immunostrip assay, reporting a sensitivity of 95% and a specificity of 83%, which represented a major advantage over current standard diagnostic tests [25]. Although the sensitivity of 86.5% with our miniaturized protein array seems to be slightly lower, we observed a higher specificity of 97% compared to the immunostrip assay. The results of our protein array are promising, since our pool of negative control sera also contained 60 patients’ sera with proven positive blood cultures for other bacterial pathogens and fungi. The high specificity (96%) of the IHA test in our healthy Thai control cohort is surprising in the context of the previous literature. It seems possible that this cohort from Ubon Ratchathani experienced a lower exposure to Burkholderia spp. than previously published cohorts.

Most signals and the highest signal intensities in positive sera were obtained from proteins involved in protein folding and stabilization or detoxification, like the two GroEL homologs (BPSL2697 and BPSS0477), two GroES homologs (BPSL2698 and BPSS0476), and the alkyl hydroperoxide reductase BPSL2096, respectively [25, 41]. Heat-shock proteins such as GroEL are generally not considered to be good serodiagnostic candidates, because cross-reactivities between different bacterial species have been described. However, the results described by Felgner *et al*. (2009) were confirmed and showed that the heat-shock protein GroEL (BPSL2697) is the most significantly differentially reactive antigen [25]. With respect to the GroEL homologs BPSL2697 and BPSS0477, and the GroES homologs BPSL2698 and BPSS0476, we cannot exclude the induction of cross reacting IgG with specificity for common epitopes, since both homolog pairs show high identities with each other (84.3% and 79.2%, respectively). Many melioidosis sera reacted with both homologs, but we also found sera recognizing only one of the paralogs, implying an induction of specific antibodies against one of these antigens. Both GroEL and GroES are known to induce strong humoral and cellular immune responses in a variety of bacterial infections [42–45], and have been proposed as universal vaccine candidates [46]. The alkyl hydroperoxide reductase BPSL2096 was another protein found to be highly antigenic. An upregulation of AhpC homologs was shown for other intracellular species as part of the response to host oxidative stress, and homologs of AhpC were previously shown to be highly immunogenic [47–49].

The immune response to the facultatively intracellular *B*. *pseudomallei* is the subject of intensive research, with many questions still unanswered [50–52]. In fact, both cell-mediated and humoral immune responses play important roles in protection against melioidosis [29, 53]. The validation of our microarray with sera taken from patients at defined time points after admission (week 0, 12 and 52 *p*.*a*.) revealed significant differences between antibodies with different specificities. To the best of our knowledge, this is the first report of short- and long-term human antibodies to *B*. *pseudomallei* protein antigens. A significant, strong antibody response was demonstrated for seven *B*. *pseudomallei* proteins (group 1 antigens), even 52 weeks after infection. Among them are GroEL and GroES homologs, the alkyl hydroperoxide reductase BPSL2096, and the outer membrane protein BPSL2522. The outer membrane protein BPSL2522 was shown to be protective in a murine model of disease and to induce a cellular and humoral immune response [54]. Future investigations will show, if group 1 antigens are also useful as markers to detect previous infections in e.g. epidemiological studies. Significant array signals to group 2 antigens could be an indication of a more recent *B*. *pseudomallei* infection, since these antigens were mainly recognized in sera of weeks 0 and 12 *p*.*a*., but rarely seen in sera drawn 52 weeks after infection. Interestingly, among this group are two effector proteins (BPSS1525 and BPSS1532) of the type III secretion system cluster 3 (TTSS3), which are essential for full virulence in murine models [55–57]. A previous study demonstrated that BPSS1525 (BopE) could induce specific CD4^+^ T cells but not CD8^+^ cells [58]. Further short-term antibody responses were found for flagellin (BPSL3319) and FlgK (BPSL0280), two proteins that are part of the flagellar apparatus. Flagellin is important for full virulence in mice and is likely to evoke a T cell response [40, 59, 60]. The flagellar hook-associated protein FlgK was found to elicit a very short-term antibody response of less than 12 weeks. This is in contrast to the study by Suwannasaen *et al*., who describe that FlgK was mainly recognized in sera of recovered melioidosis patients by using the protein array technology of Felgner *et al*. [40]. But more data are needed to validate the proteins identified as possible early and late antigens in melioidosis diagnostics.

In summary, the protein array technology used in this study enables a comprehensive recognition of *B*. *pseudomallei*-specific antibody responses in melioidosis. It allows multiplex antigen detection in a miniaturized and automated fashion replacing the traditional “one antigen at a time” method [23, 61–64]. Generally, our technical approach allows non-proteinogenic antigens, such as the various polysaccharide antigens described, to be included in the protein microarray format. Our method can also be applied for the analysis of *B*. *pseudomallei* protein expression *in vivo* using experimental animal models and can be used to elucidate the exposure to *B*. *pseudomallei* in humans and animals in epidemiological studies. Future multicenter studies are needed to determine the true sensitivity and specificity of this protein array as a diagnostic tool in different parts of the world. We are aware that the protein array technology presented might not be affordable in remote rural endemic areas. However, results of future multicenter protein array studies might finally translate into multiple antigen based point of care (POC) devices such as lateral flow assays, which should be applicable in low-resource tropical settings.

## Supporting Information

S1 Checklist STARD checklist. (DOCX)

S1 Fig SDS-PAGE of all recombinant proteins after purification. Three μg per protein were applied and gels were stained by Coomassie Brilliant Blue G-250. Strep—*Strep*-tag, His–His-tag, M–protein ladder.
(PDF)

S2 Fig Workflow of protein array detection. Arraystrips were incubated with the respective blood sera or plasmas and developed as described in Material and Methods. Afterwards, the Arraystrips were read out by a CCD camera using the ArrayMate from Alere Technologies GmbH. The pictures were quantified and normalized by IconoClust software. The whole protocol from incubation of sera/plasmas to final analysis takes about 2 hours.
(PDF)

S3 Fig Heatmap of probing a collection of melioidosis-positive sera and negative control sera. Protein arrays containing 20 *B*. *pseudomallei* recombinant proteins were probed with 356 melioidosis and non-melioidosis sera. The melioidosis-positive sera are composed of sera from patients upon admission (week 0 *p*.*a*.) and of weeks 12 *p*.*a*. and 52 week *p*.*a*. All positive sera were sampled in Ubon Ratchathani, Thailand. Negative control sera of healthy persons were sampled in endemic regions of Thailand (Ubon Ratchathani (U.R.)) or in the non-endemic regions of Bangkok (B.) Thailand and Greifswald (Germany). Additionally, further negative control sera of patients with other bacteremia or fungaemia were used from the same non-endemic region. The antigens are shown in rows with five increasing concentrations per protein, and the patient samples are represented in columns. Array signals are reflected by the intensities of the color (white to blue) inside the boxes. The heatmap was created using Multi experiment Viewer (MeV 4.9.0) from TM4 suite, USA.
(PDF)

S4 Fig Correlation of antigen recognition and applied antigen concentrations. The number of recognized antigens per group of serum depending on different spotted antigen concentrations and their medians are shown. Results for *His*-tagged antigens are not shown. Statistical analyses were performed using one-way analysis of variance (ANOVA) followed by Bonferroni correction, comparing titers of grouped melioidosis sera of weeks 0 *p*.*a*. (n = 75), 12 *p*.*a*. (n = 50), and 52 *p*.*a*. (n = 46), as well as those of non-melioidosis healthy persons (non-melioidosis, n = 125) and patients with other bacteremias/fungemias (other bac/fun, n = 60) (*p<0.05; ***p<0.001).
(PDF)

S5 Fig Identification scheme of *B*. *pseudomallei* serodiagnostic antigens using sera from melioidosis patients from Thailand and respective control sera. Arrows and corresponding numbers represent the single statistical analyses between the particular groups. Sums of all positive signals per protein and group were used for Fisher’s exact test, and *B*. *pseudomallei* antigens with *p*-values ≤0.01 were assumed to differ significantly between the groups. Results of these analyses are shown in the table of the S1 File.
(PDF)

S6 Fig Heatmap of probing a collection of melioidosis positive and negative plasmas. The plasmas (n = 50) are composed of samples from patients with acute *B*. *pseudomallei* infections (n = 25) and negative controls (n = 25) from healthy persons. All plasmas were sampled in Ubon Ratchathani, Thailand. The antigens are shown in rows with five increasing concentrations per protein, and the patient samples are represented in columns. Array signals are reflected by the intensities of the color (white to blue) inside the boxes. The heatmap was created using Multi experiment Viewer (MeV 4.9.0) from TM4 suite, USA.
(PDF)

S7 Fig Correlation of antigen recognition in melioidosis-positive and -negative plasma samples. The numbers of recognized antigens (spotted antigen concentration 0.45 mg/ml) per plasma and the respective medians are shown. Results for *His*-tagged antigens are not shown. Signals were assumed to be positive if the intensity was at least 0.3. Statistical analyses were performed using the Mann-Whitney test on melioidosis-positive plasmas (n = 25) and nonmelioidosis healthy persons (n = 25) (***p<0.001).
(PDF)

S8 Fig Comparison of antigen recognition between melioidosis-positive sera and plasma samples. The number of recognized antigens (spotted antigen concentration 0.45 mg/ml) per sera or plasma and the respective medians are shown. Results for *His*-tagged antigens are not shown. Signals were assumed to be positive if the intensity was at least 0.3. Statistical analyses were performed using the Mann-Whitney test on melioidosis positive sera (n = 75) and plasmas (n = 25). (n.s.—not significant (*p* = 0.4273)).
(PDF)

S9 Fig Signal intensities of all *B*. *pseudomallei* proteins recognized by 20 melioidosis-positive blood sera and corresponding blood plasmas. The antigens and controls applied at five different dilutions are shown on the x-axes and measured signal intensities on the y-axes. Blue bars represent the intensities measured in sera, and green bars show intensities for the respective plasmas. Corresponding samples were drawn at the same time point. For a higher resolution zoom in.
(PDF)

S1 Table Bacterial strains and plasmids used in this study. (PDF)

S2 Table Primers, plasmids and restriction enzymes used for cloning protein encoding genes and for recombinant protein expression. (PDF)

S3 Table Microbial species identified in blood cultures of non-melioidosis patients with other bacteremia and fungaemia. (PDF)

S1 File Significantly recognized antigens from *B*. *pseudomallei*. Columns represent the corresponding comparison from S5 Fig and rows show the spotted *B*. *pseudomallei* antigens. All blue stained boxes mark significantly recognised *B*. *pseudomallei* antigens in the respective comparison. Statistical analysis was performed using the Fisher's exact test. *p* values smaller than or equal 0.01 were assumed to differ significantly between the respective sera group. Shown are *p* values for every antigen spotted with a concentration of 0.45 mg/ml.
(PPTX)

S2 File Signal intensities of all blood sera and plasmas. (XLSX)

S3 File **Signal intensities of grouped antigens inducing a long-term antibody response (A) and a short-term antibody response (B) of all tested sera**. Sera of patients were drawn upon admission at week 0 (n = 75), 12 (n = 50) and 52 (n = 46) weeks *p*.*a*. Shown are signal intensities of the respective antigen obtained from the corresponding sera. Figures include only data of antigens found to be significantly recognized by melioidosis-positive sera. The last diagram of group **A** and **B** antigens shows the percentage of sera of the respective cohort recognizing single antigens. Red lines represent the used cut-off (≥ 0.3) for the analyses of the protein arrays.
(PDF)

S1 Flow Diagram STARD flow diagram. (PDF)
