# Supplementary material for: Rapid and Sensitive Multiplex Detection of Burkholderia pseudomallei-Specific Antibodies in Melioidosis Patients Based on a Protein Microarray Approach
Source: PLoS Negl Trop Dis. 2016 Jul 18;10(7):e0004847. doi: 10.1371/journal.pntd.0004847 (PMC4948818; doi:10.1371/journal.pntd.0004847)
Supplement: S1 Fig — Three μg per protein were applied and gels were stained by Coomassie Brilliant Blue G-250. Strep—Strep-tag, His–His-tag, M–protein ladder. (PDF) [file pntd.0004847.s002.pdf]

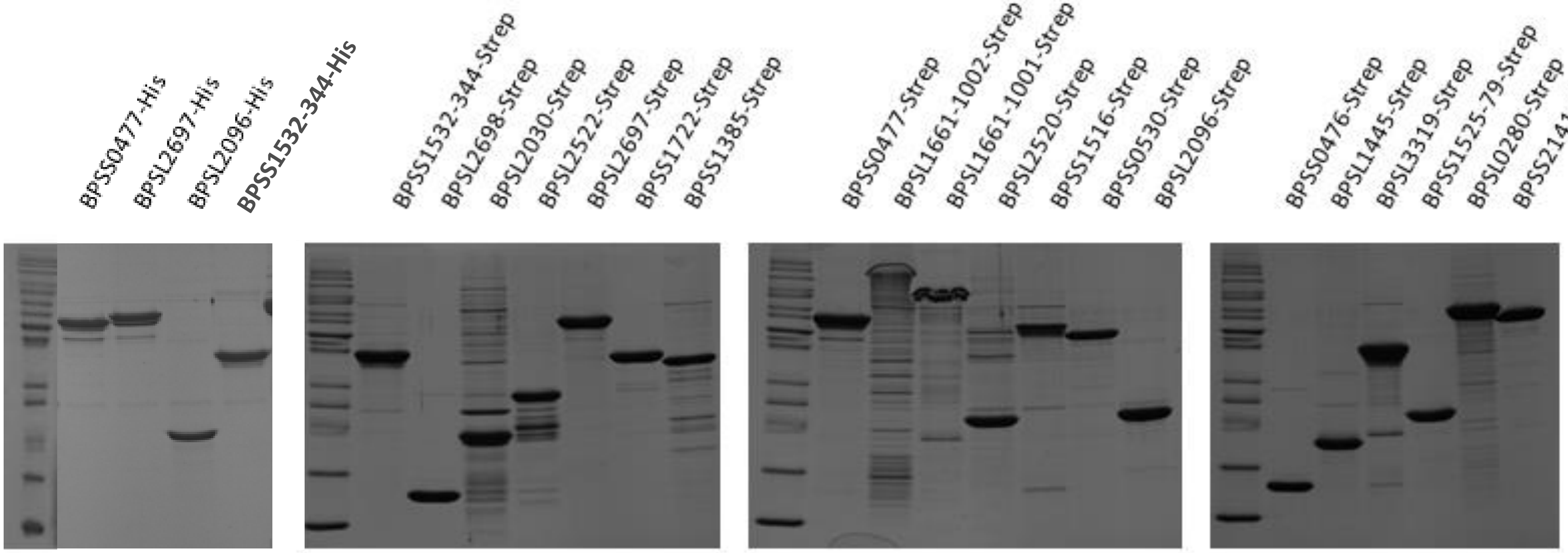
BPSS0477-His
BPSL2697-His
BPSL2096-His
BPSS1532-344-His
BPSS1532-344-Strep
BPSL2698-Strep
BPSL2030-Strep
BPSL2522-Strep
BPSL2697-Strep
BPSS1722-Strep
BPSS1385-Strep
BPSS0477-Strep
BPSL1661-1002-Strep
BPSL1661-1001-Strep
BPSL2520-Strep
BPSS1516-Strep
BPSS0530-Strep
BPSL2096-Strep
BPSS0476-Strep
BPSL1445-Strep
BPSL3319-Strep
BPSS1525-79-Strep
BPSL0280-Strep
BPSS2141-Strep
