# Supplementary material for: Rapid and Sensitive Multiplex Detection of Burkholderia pseudomallei-Specific Antibodies in Melioidosis Patients Based on a Protein Microarray Approach
Source: PLoS Negl Trop Dis. 2016 Jul 18;10(7):e0004847. doi: 10.1371/journal.pntd.0004847 (PMC4948818; doi:10.1371/journal.pntd.0004847)
Supplement: S2 Fig — Arraystrips were incubated with the respective blood sera or plasmas and developed as described in Material and Methods. Afterwards, the Arraystrips were read out by a CCD camera using the ArrayMate from Alere Technologies GmbH. The pictures were quantified and normalized by IconoClust software. The whole protocol from incubation of sera/plasmas to final analysis takes about 2 hours. (PDF) [file pntd.0004847.s003.pdf]

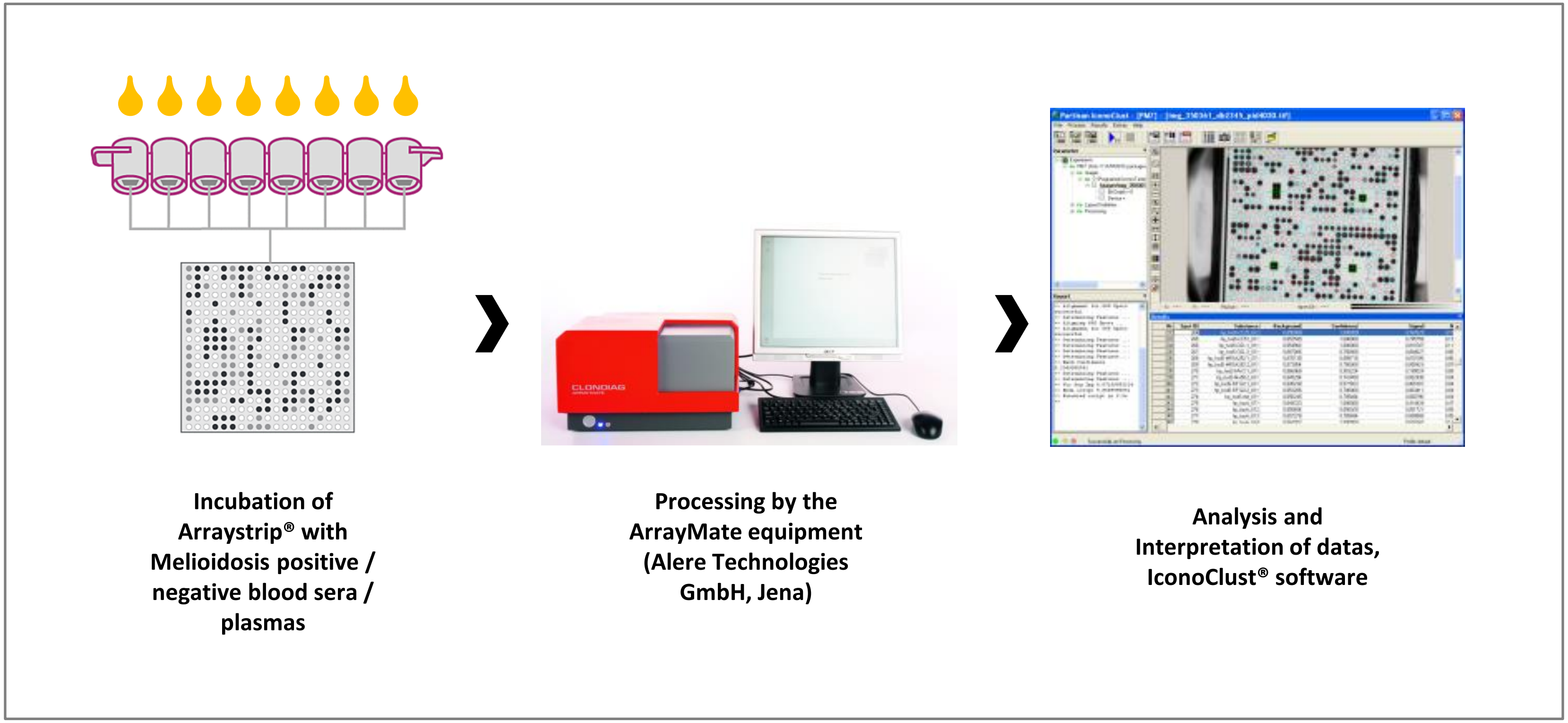
CLONDIAG
Incubation of Arraystrip® with Melioidosis positive / negative blood sera / plasmas
Processing by the ArrayMate equipment (Alere Technologies GmbH, Jena)
Analysis and Interpretation of datas, IconoClust® software
