# Supplementary figures and images for: Rapid and Sensitive Multiplex Detection of Burkholderia pseudomallei-Specific Antibodies in Melioidosis Patients Based on a Protein Microarray Approach
Source: PLoS Negl Trop Dis. 2016 Jul 18;10(7):e0004847. doi: 10.1371/journal.pntd.0004847 (PMC4948818; doi:10.1371/journal.pntd.0004847)

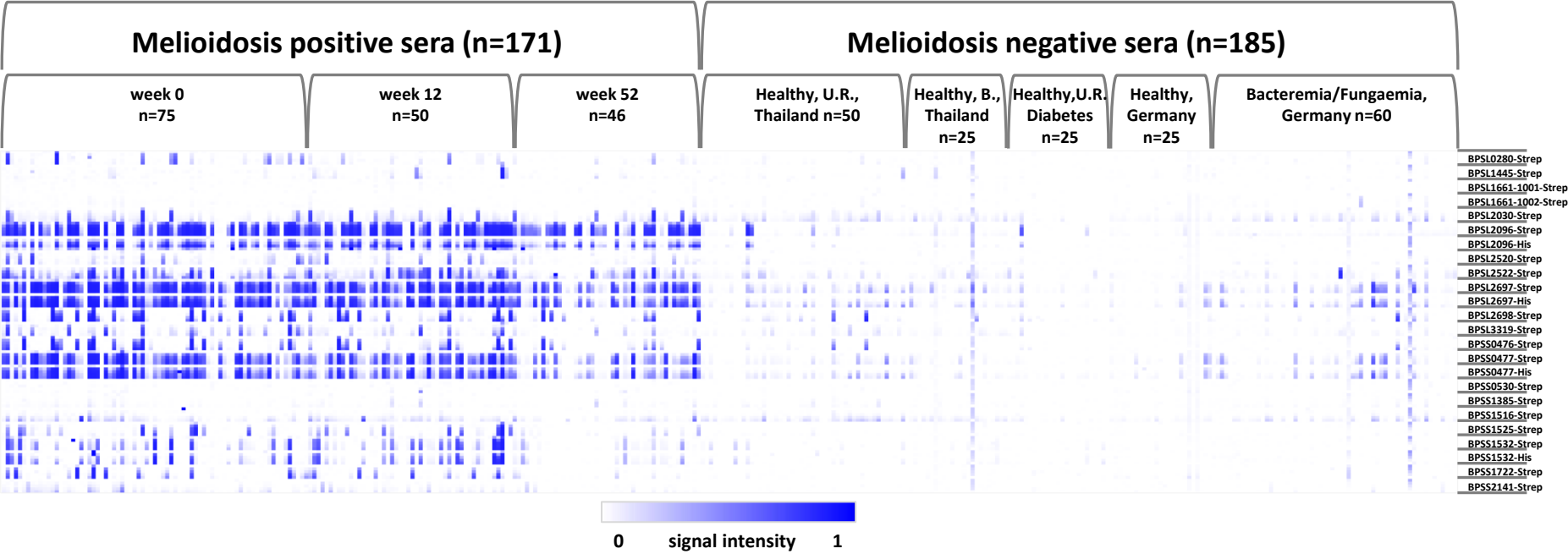

Supplement: S3 Fig — Protein arrays containing 20 B. pseudomallei recombinant proteins were probed with 356 melioidosis and non-melioidosis sera. The melioidosis-positive sera are composed of sera from patients upon admission (week 0 p.a.) and of weeks 12 p.a. and 52 week p.a. All positive sera were sampled in Ubon Ratchathani, Thailand. Negative control sera of healthy persons were sampled in endemic regions of Thailand (Ubon Ratchathani (U.R.)) or in the non-endemic regions of Bangkok (B.) Thailand and Greifswald (Germany). Additionally, further negative control sera of patients with other bacteremia or fungaemia were used from the same non-endemic region. The antigens are shown in rows with five increasing concentrations per protein, and the patient samples are represented in columns. Array signals are reflected by the intensities of the color (white to blue) inside the boxes. The heatmap was created using Multi experiment Viewer (MeV 4.9.0) from TM4 suite, USA. (PDF) [file pntd.0004847.s004.pdf]

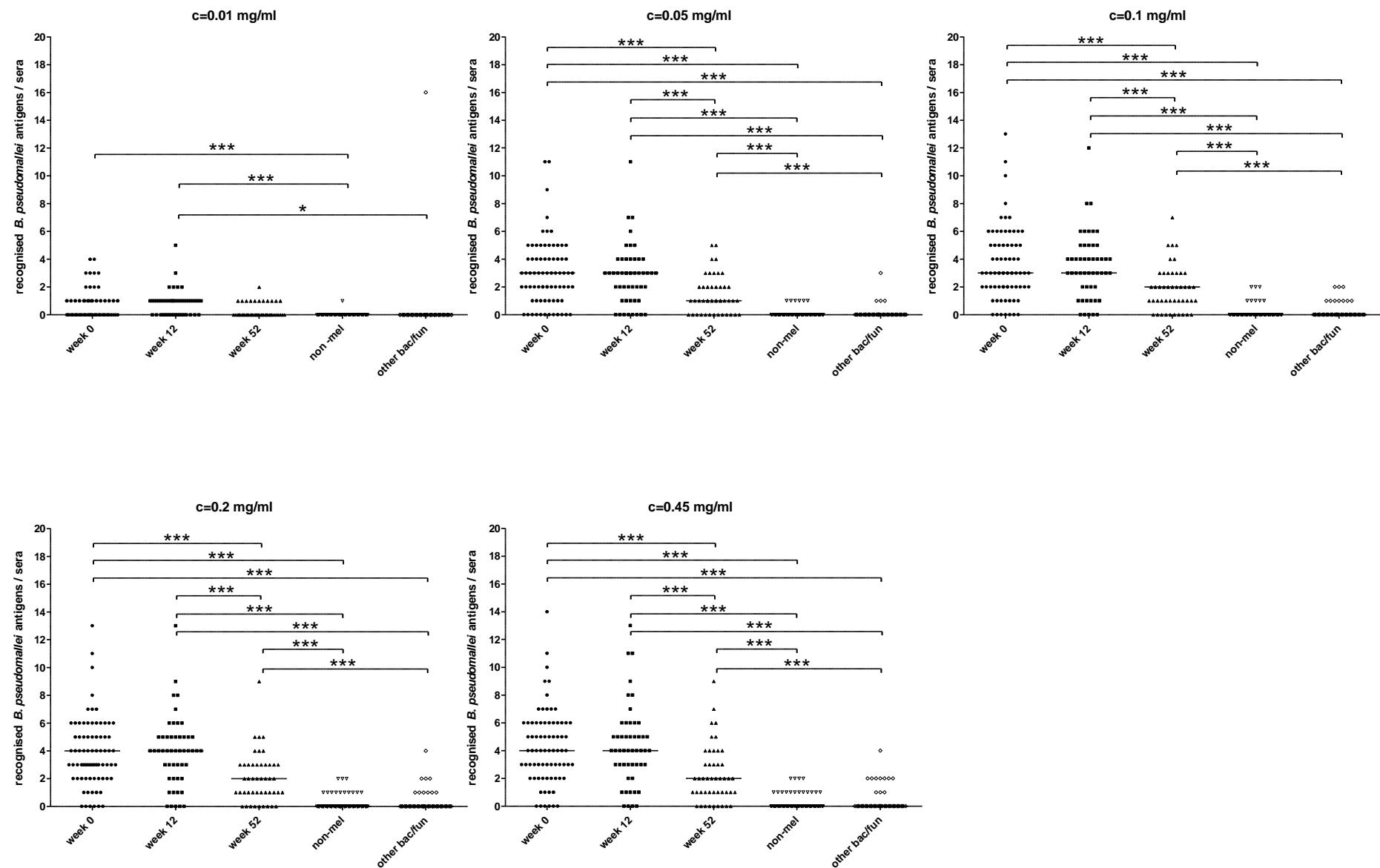

Supplement: S4 Fig — The number of recognized antigens per group of serum depending on different spotted antigen concentrations and their medians are shown. Results for His-tagged antigens are not shown. Statistical analyses were performed using one-way analysis of variance (ANOVA) followed by Bonferroni correction, comparing titers of grouped melioidosis sera of weeks 0 p.a. (n = 75), 12 p.a. (n = 50), and 52 p.a. (n = 46), as well as those of non-melioidosis healthy persons (non-melioidosis, n = 125) and patients with other bacteremias/fungemias (other bac/fun, n = 60) (*p<0.05; ***p<0.001). (PDF) [file pntd.0004847.s005.pdf]

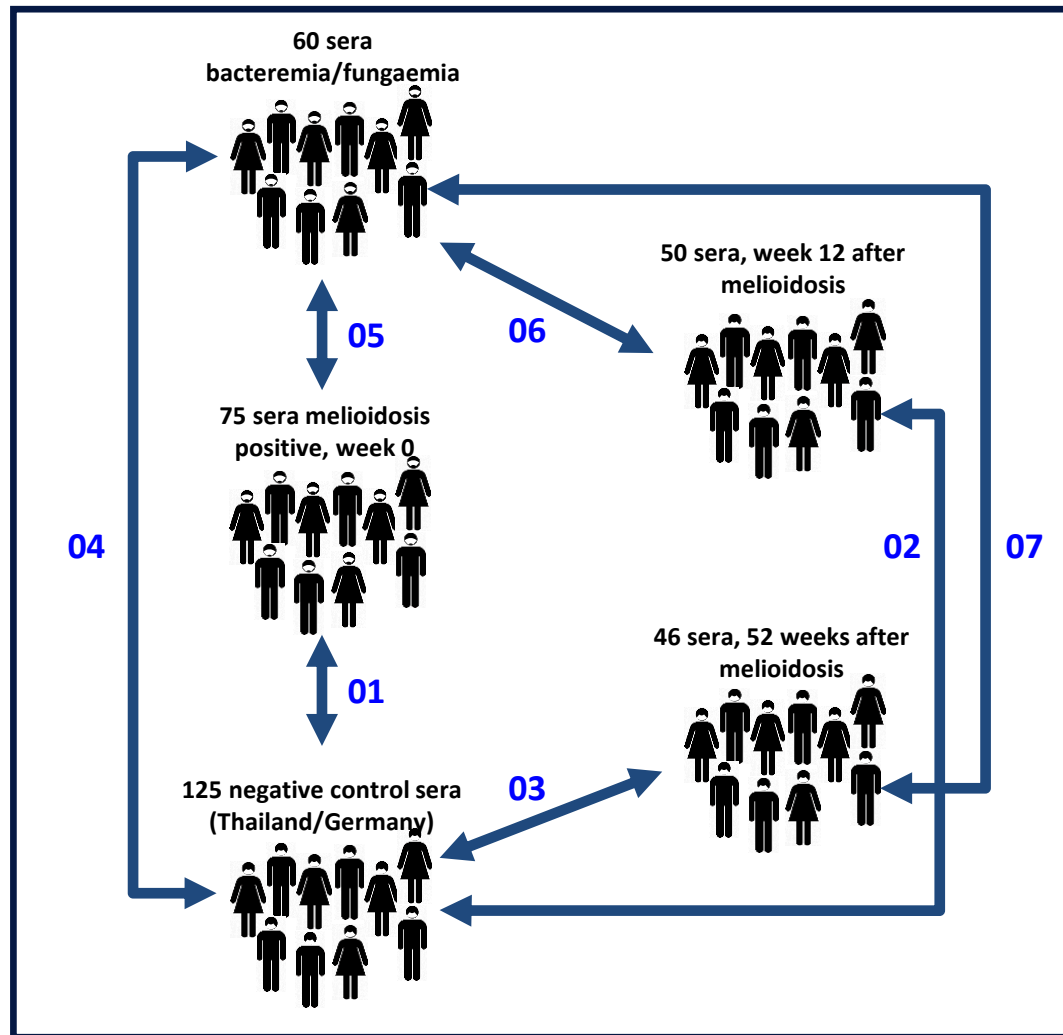

Supplement: S5 Fig — Arrows and corresponding numbers represent the single statistical analyses between the particular groups. Sums of all positive signals per protein and group were used for Fisher’s exact test, and B. pseudomallei antigens with p-values ≤0.01 were assumed to differ significantly between the groups. Results of these analyses are shown in the table of the S1 File. (PDF) [file pntd.0004847.s006.pdf]

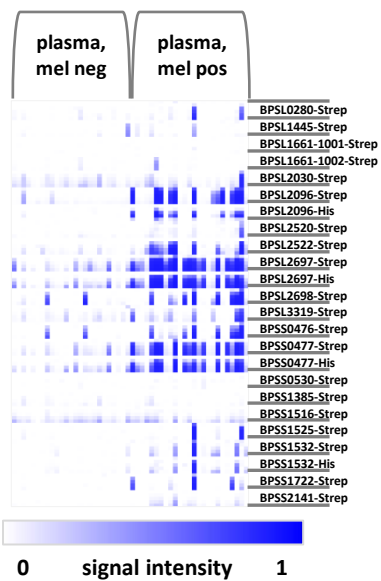

Supplement: S6 Fig — The plasmas (n = 50) are composed of samples from patients with acute B. pseudomallei infections (n = 25) and negative controls (n = 25) from healthy persons. All plasmas were sampled in Ubon Ratchathani, Thailand. The antigens are shown in rows with five increasing concentrations per protein, and the patient samples are represented in columns. Array signals are reflected by the intensities of the color (white to blue) inside the boxes. The heatmap was created using Multi experiment Viewer (MeV 4.9.0) from TM4 suite, USA. (PDF) [file pntd.0004847.s007.pdf]

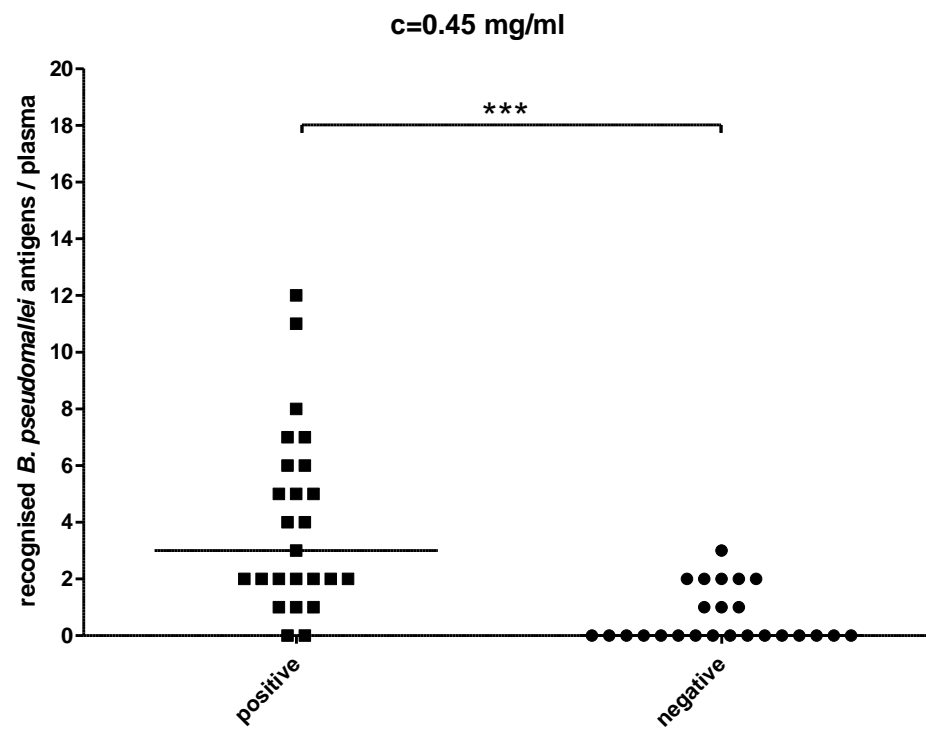

Supplement: S7 Fig — The numbers of recognized antigens (spotted antigen concentration 0.45 mg/ml) per plasma and the respective medians are shown. Results for His-tagged antigens are not shown. Signals were assumed to be positive if the intensity was at least 0.3. Statistical analyses were performed using the Mann-Whitney test on melioidosis-positive plasmas (n = 25) and nonmelioidosis healthy persons (n = 25) (***p<0.001). (PDF) [file pntd.0004847.s008.pdf]

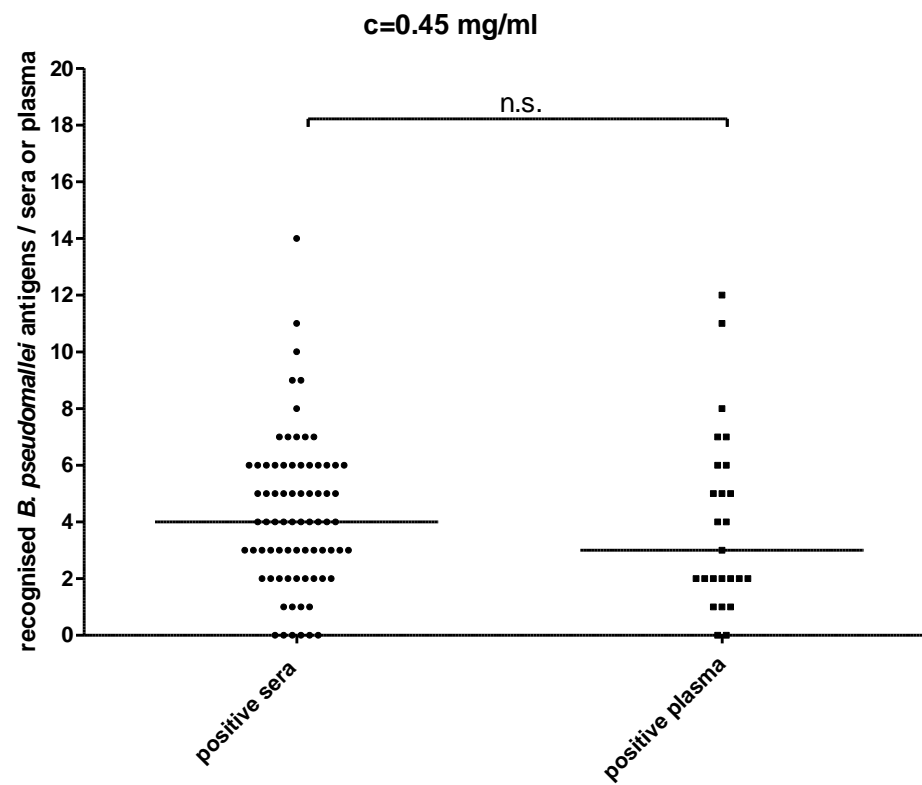

Supplement: S8 Fig — The number of recognized antigens (spotted antigen concentration 0.45 mg/ml) per sera or plasma and the respective medians are shown. Results for His-tagged antigens are not shown. Signals were assumed to be positive if the intensity was at least 0.3. Statistical analyses were performed using the Mann-Whitney test on melioidosis positive sera (n = 75) and plasmas (n = 25). (n.s.—not significant (p = 0.4273)). (PDF) [file pntd.0004847.s009.pdf]

A

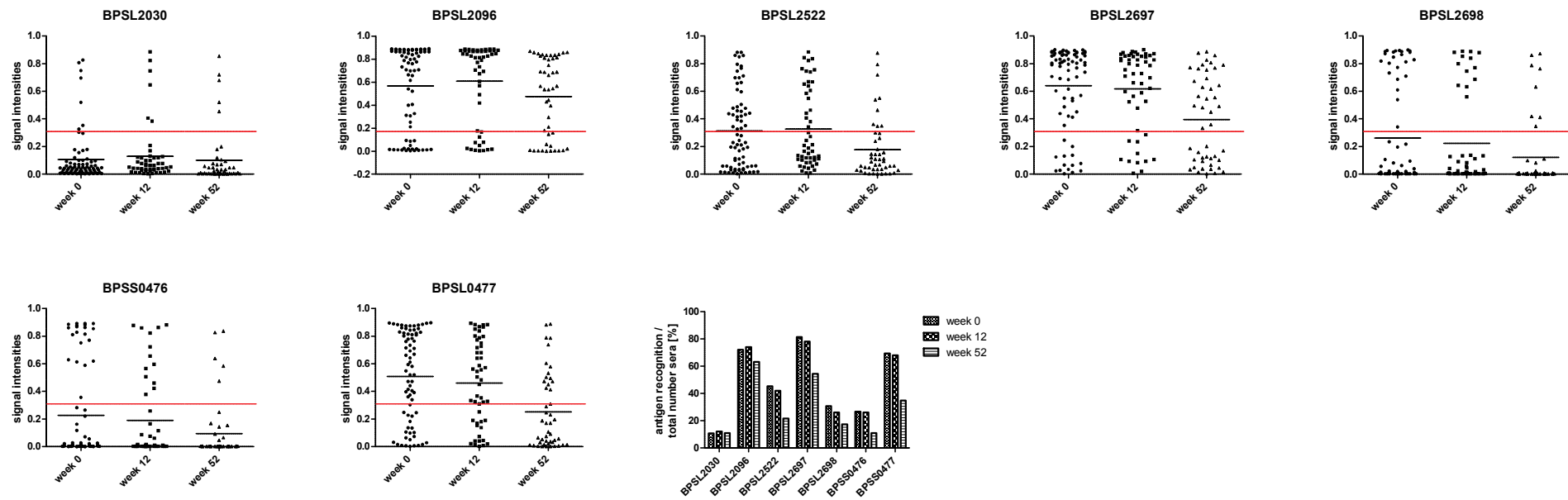

B

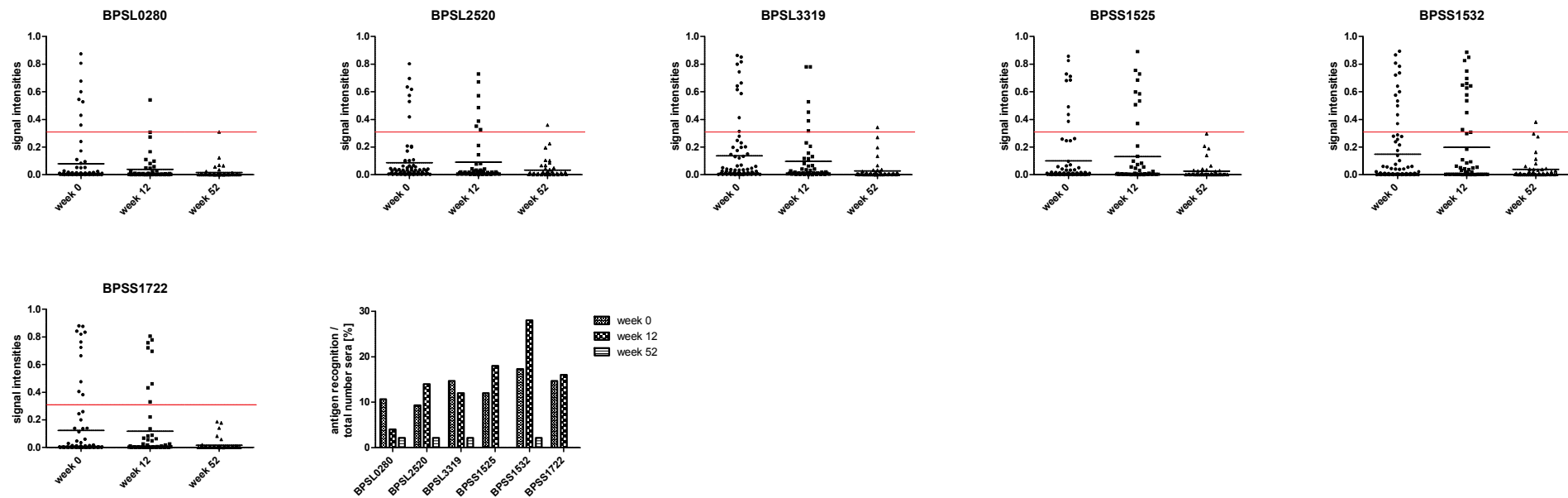

Supplement: S3 File — Signal intensities of grouped antigens inducing a long-term antibody response (A) and a short-term antibody response (B) of all tested sera. Sera of patients were drawn upon admission at week 0 (n = 75), 12 (n = 50) and 52 (n = 46) weeks p.a. Shown are signal intensities of the respective antigen obtained from the corresponding sera. Figures include only data of antigens found to be significantly recognized by melioidosis-positive sera. The last diagram of group A and B antigens shows the percentage of sera of the respective cohort recognizing single antigens. Red lines represent the used cut-off (≥ 0.3) for the analyses of the protein arrays. (PDF) [file pntd.0004847.s016.pdf]
