# Supplementary material for: Rapid and Sensitive Multiplex Detection of Burkholderia pseudomallei-Specific Antibodies in Melioidosis Patients Based on a Protein Microarray Approach
Source: PLoS Negl Trop Dis. 2016 Jul 18;10(7):e0004847. doi: 10.1371/journal.pntd.0004847 (PMC4948818; doi:10.1371/journal.pntd.0004847)

serum/plasma 1

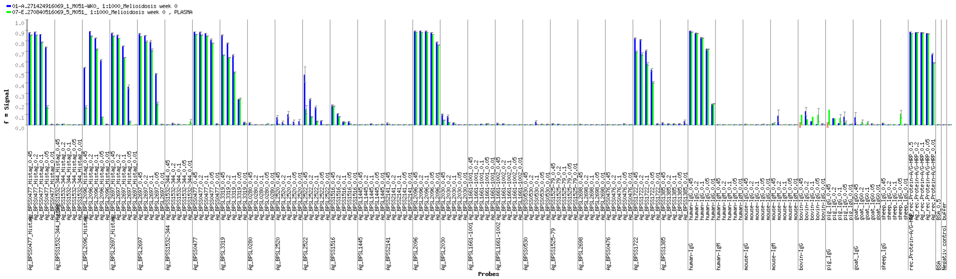

serum/plasma 2

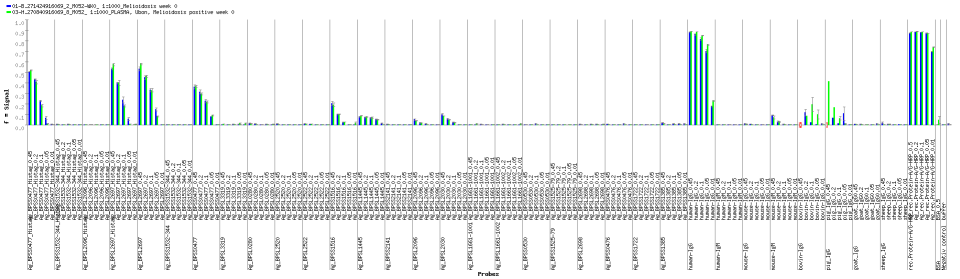

serum/plasma 3

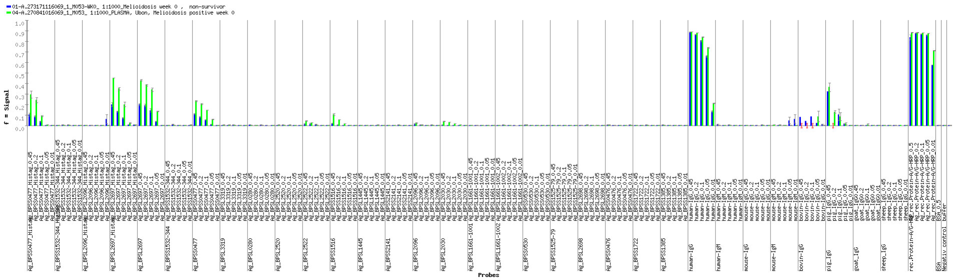

serum/plasma 4

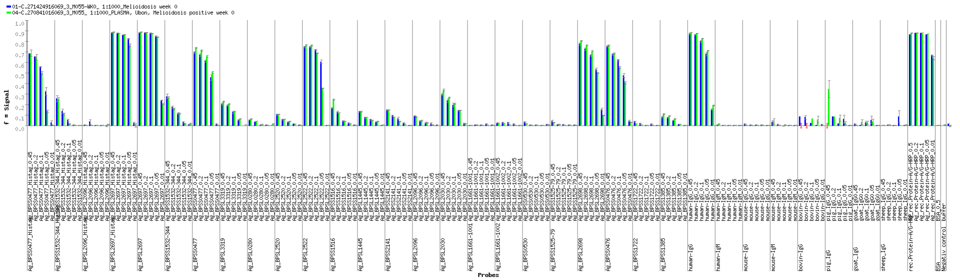

serum/plasma 5

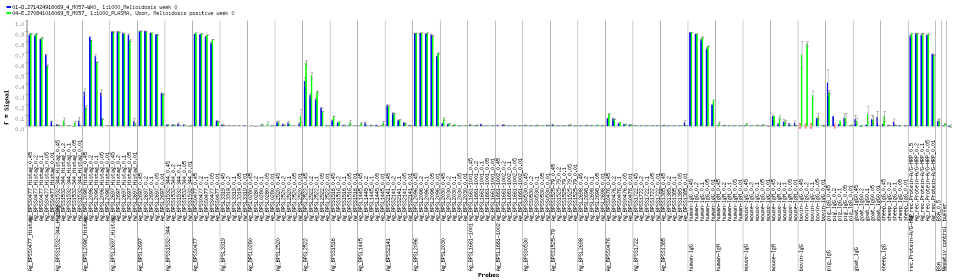

serum/plasma 6

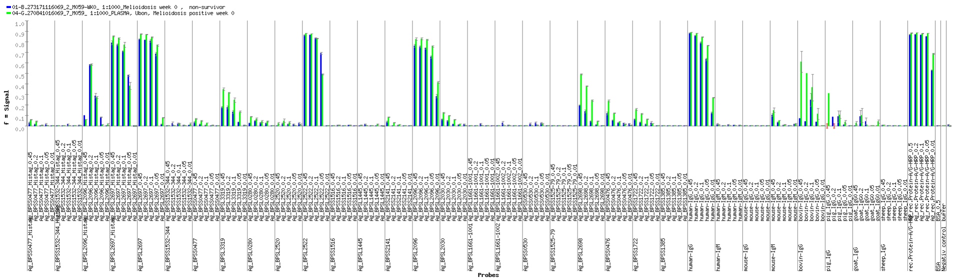

serum/plasma 7

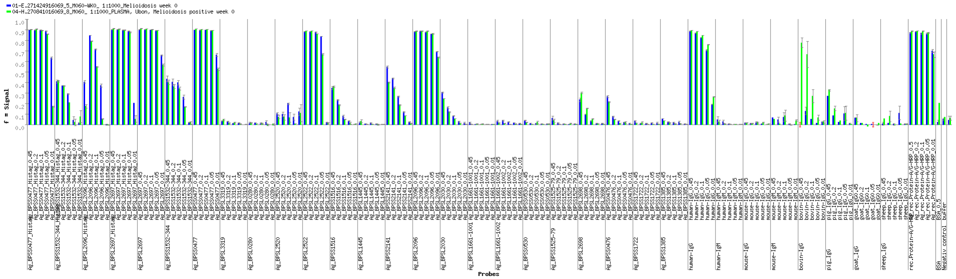

serum/plasma 8

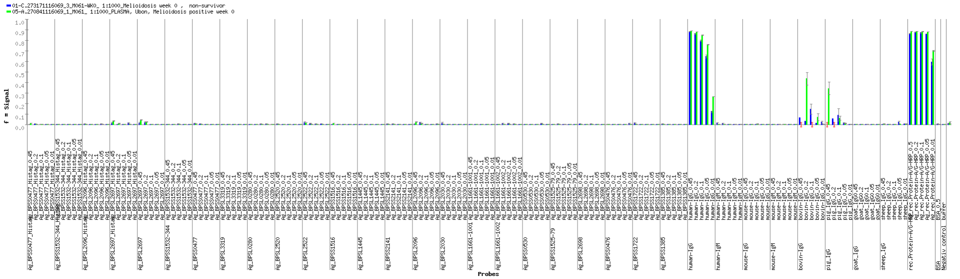

serum/plasma 9

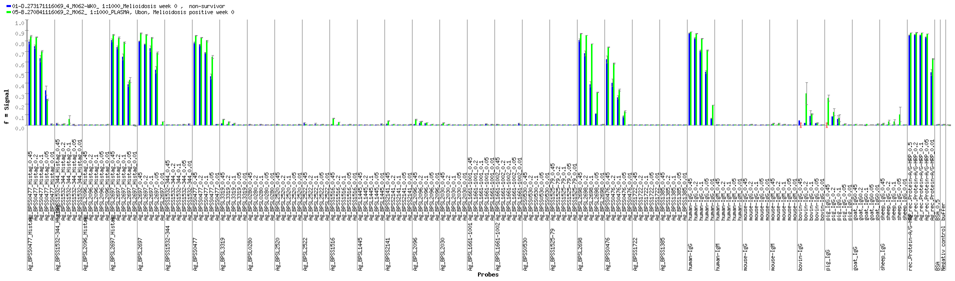

serum/plasma 10

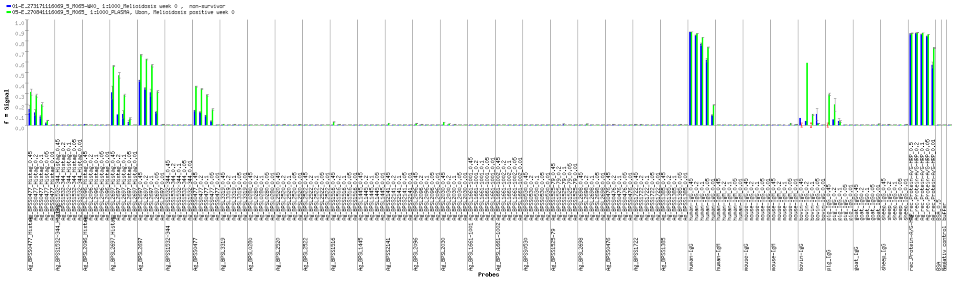

serum/plasma 11

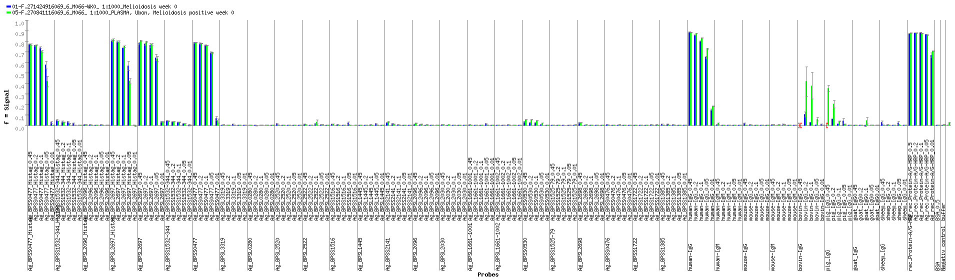

serum/plasma 12

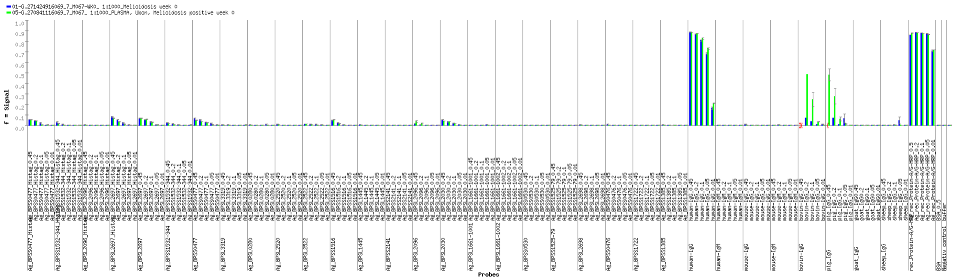

serum/plasma 13

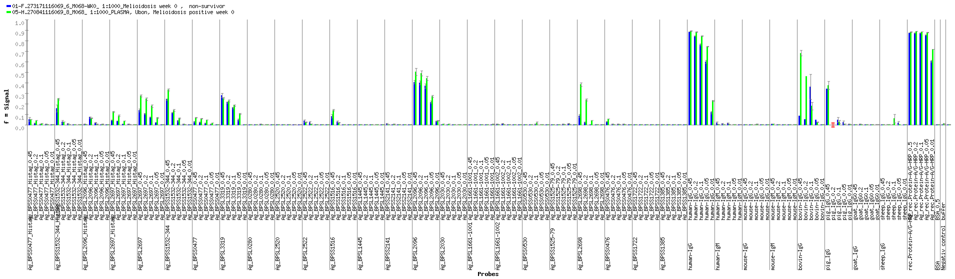

serum/plasma 14

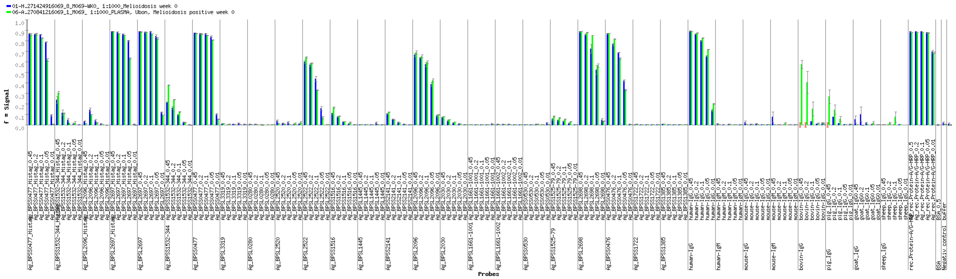

serum/plasma 15

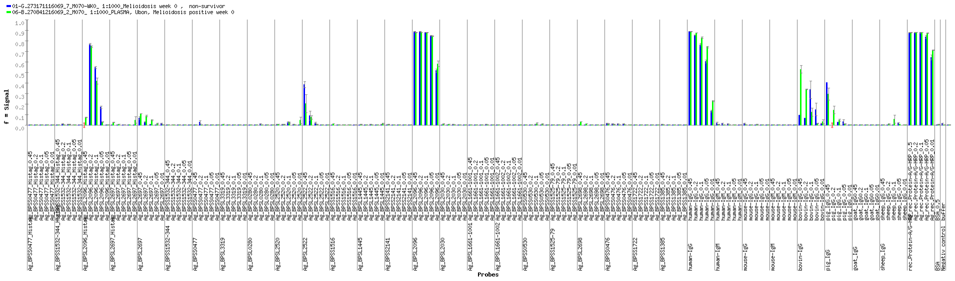

serum/plasma 16

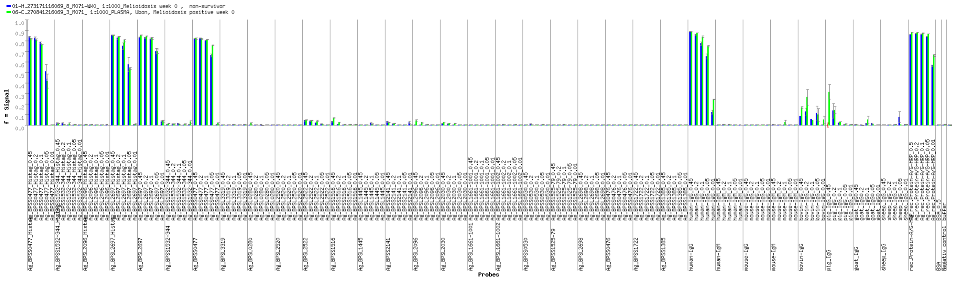

serum/plasma 17

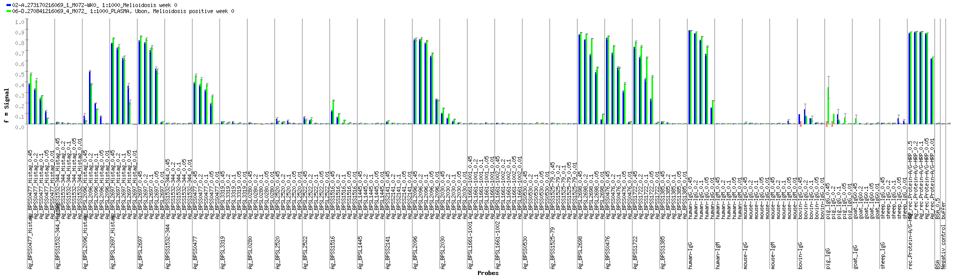

serum/plasma 18

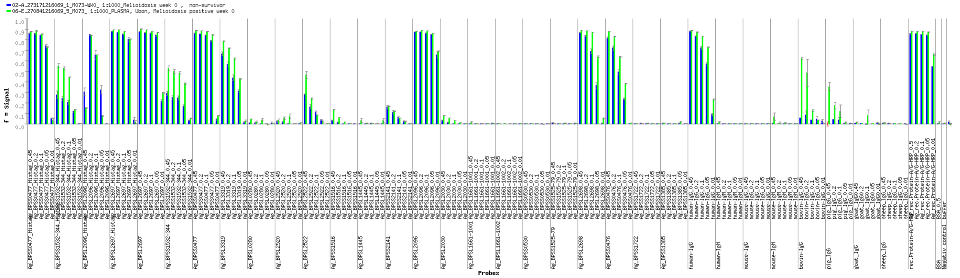

serum/plasma 19

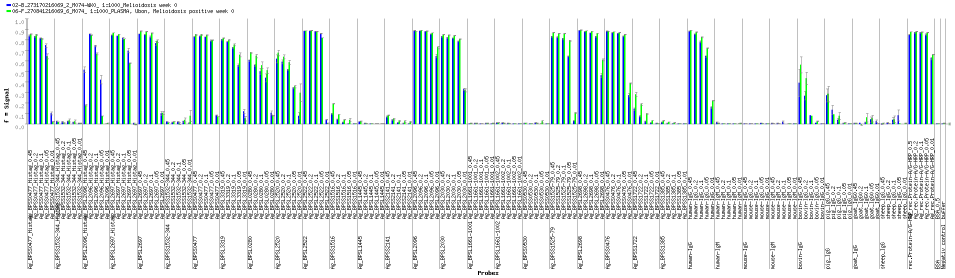

serum/plasma 20

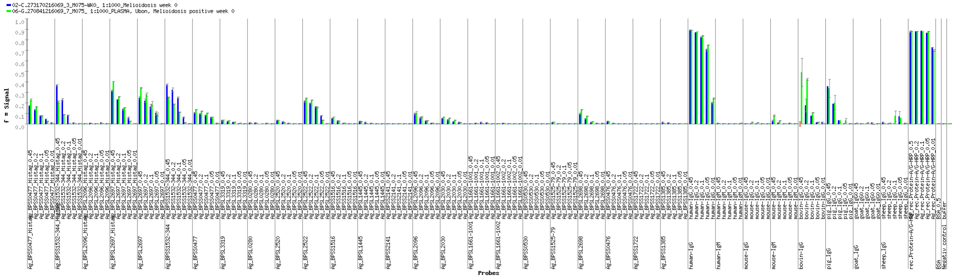

Supplement: S9 Fig — The antigens and controls applied at five different dilutions are shown on the x-axes and measured signal intensities on the y-axes. Blue bars represent the intensities measured in sera, and green bars show intensities for the respective plasmas. Corresponding samples were drawn at the same time point. For a higher resolution zoom in. (PDF) [file pntd.0004847.s010.pdf]
