# Supplementary material for: Rapid and Sensitive Multiplex Detection of Burkholderia pseudomallei-Specific Antibodies in Melioidosis Patients Based on a Protein Microarray Approach
Source: PLoS Negl Trop Dis. 2016 Jul 18;10(7):e0004847. doi: 10.1371/journal.pntd.0004847 (PMC4948818; doi:10.1371/journal.pntd.0004847)
Supplement: S1 Table — (PDF) [file pntd.0004847.s011.pdf]

Strain	Characteristics	Reference
Escherichia coli		
DH5a	cloning host	Invitrogen, Life technologies, Darmstadt, Germany
BL21 (DE3) pLysS	expression strain	Invitrogen, Life technologies, Darmstadt, Germany
Burkholderia pseudomallei		
K96243	*B. pseudomallei* strain K96243 wild type	Holden et al. 2004
Plasmids		
pPR-IBA1	expression vector, *Strep* tag	IBM GmbH, Goetingen, Germany
pASK-IBA33plus	expression vector, *His* tag	IBM GmbH, Goetingen, Germany
