# Supplementary material for: Rapid and Sensitive Multiplex Detection of Burkholderia pseudomallei-Specific Antibodies in Melioidosis Patients Based on a Protein Microarray Approach
Source: PLoS Negl Trop Dis. 2016 Jul 18;10(7):e0004847. doi: 10.1371/journal.pntd.0004847 (PMC4948818; doi:10.1371/journal.pntd.0004847)
Supplement: S2 Table — (PDF) [file pntd.0004847.s012.pdf]

Antigen	Used locus name for protein array	Primer forward 5´-3´	Primer reverse 5´-3´	Restriction enzyme	Plasmid	tag
**BPSL0280**	BPSL0280	ATGGTACGTCTCAAATGTCCAATACCCTCATGAACCTCGG	ATGGTACGTCTCAGCGCTGTTGAAGAGACCGAGCACGGTC	BsmBI	pPR-IBA1	C-terminal Strep-tag
**BPSL1445**	BPSL1445	ATGGTAGGTCTCAAATGGGTTGCACGACGACGCCGGA	ATGGTAGGTCTCAGCGCTGATCTTCAGCTTCGTGACCTTCG	BsaI	pPR-IBA1	C-terminal Strep-tag
**BPSL1661**	BPSL1661-1001	ATGGTAGGTCTCAAATGAATACGAAGATGTTGCTGATAAGAG	ATGGTAGGTCTCAGCGCTACCGCCGCTGCTGTCCGCC	BsaI	pPR-IBA1	C-terminal Strep-tag
**BPSL1661**	BPSL1661-1002	ATGGTAGGTCTCAAATGTGGACGACGACGTTGACGAGC	ATGGTAGGTCTCAGCGCTGGGCGTGAGCGCGGTCAGC	BsaI	pPR-IBA1	C-terminal Strep-tag
**BPSL2030**	BPSL2030	ATGGTAGGTCTCAAATGCAGACCAGCCCGCTCACGGG	ATGGTAGGTCTCAGCGCTCCAGCCGAGCGTGACGTTCAG	BsaI	pPR-IBA1	C-terminal Strep-tag
**BPSL2096**	BPSL2096	ATGGTAGGTCTCAAATGGGCGACGCGAAACAGGCGATC	ATGGTAGAAGACAAGCGCTCAGCGTCGCGCCGCCGATC	BbsI	pPR-IBA1	C-terminal Strep-tag
**BPSL2096**	BPSL2096	ATGGTAGGTCTCAAATGGGCGACGCGAAACAGGCGATC	ATGGTAGAAGACAAGCGCTCAGCGTCGCGCCGCCGATC	BbsI	pASK-IBA33plus	C-terminal His-tag
**BPSL2520**	BPSL2520	ATGGTAGGTCTCAAATGCAATCGCTGTCGAACCAGACTTC	ATGGTAGGTCTCAGCGCTCTTGCCCGGCTTGACCGCCG	BsaI	pPR-IBA1	C-terminal Strep-tag
**BPSL2522**	BPSL2522	ATGGTAGGTCTCAAATGCAGTCGGTGCCGGCGTCGC	ATGGTAGGTCTCAGCGCTCTGCGCCGGAACGGTCGTCT	BsaI	pPR-IBA1	C-terminal Strep-tag
**BPSL2697**	BPSL2697	ATGGTAGGTCTCAAATGGCAGCTAAAGACGTCGTATTCGG	ATGGTAGGTCTCAGCGCTCATGTCCATGCCCATGCCGCC	BsaI	pPR-IBA1	C-terminal Strep-tag
**BPSL2697**	BPSL2697	ATGGTAGGTCTCAAATGGCAGCTAAAGACGTCGTATTCGG	ATGGTAGGTCTCAGCGCTCATGTCCATGCCCATGCCGCC	BsaI	pASK-IBA33plus	C-terminal His-tag
**BPSL2698**	BPSL2698	ATGGTAGGTCTCAAATGAACCTTCGTCCGTTGCACGATC	ATGGTAGGTCTCAGCGCTCTTAGCGTTGACCACGGCCATG	BsaI	pPR-IBA1	C-terminal Strep-tag
**BPSL3319**	BPSL3319	ATGGTAGAAGACAAAATGCTCGGAATCAACAGCAACATTAAC	ATGGTAGAAGACAAGCGCTTTGCAGGAGCTTCAGCACTTGC	BbsI	pPR-IBA1	C-terminal Strep-tag
**BPSS0476**	BPSS0476	ATGGTAGGTCTCAAATGAGCCTACGCCCGCTACACGAT	ATGGTAGGTCTCAGCGCTGGAATGTACGACTGCGACGATG	BsaI	pPR-IBA1	C-terminal Strep-tag
**BPSS0477**	BPSS0477	ATGGTAGGTCTCAAATGGCAGCGAAAGAGATCATTTTCCAC	ATGGTAGGTCTCAGCGCTGAAATCGAAGCCCGGGCCGC	BsaI	pASK-IBA33plus	C-terminal His-tag

Table S2. Continued.

Antigen	Used locus name for protein array	Primer forward 5´-3´	Primer reverse 5´-3´	Restriction enzyme	Plasmid	tag
**BPSS0477**	BPSS0477	ATGGTAGGTCTCAAATGGCAGCGAAAGAGATCATTTTCCAC	ATGGTAGGTCTCAGCGCTGAAATCGAAGCCCGGGCCGC	BsaI	pPR-IBA1	C-terminal Strep-tag
**BPSS0530**	BPSS0530	ATGGTAGGTCTCAAATGAGTAGTCTGCCGGTAGGACCG	ATGGTAGGTCTCAGCGCTCGCCACCTTGCCGTCTCTCAG	BsaI	pPR-IBA1	C-terminal Strep-tag
**BPSS1385**	BPSS1385	ATGGTAGGTCTCAAATGTTGGAGCACGGCGTCATGAAAAT	ATGGTAGGTCTCAGCGCTGCCAAGGCCGGCGACGTATTG	BsaI	pPR-IBA1	C-terminal Strep-tag
**BPSS1516**	BPSS1516	ATGGTAGGTCTCAAATGAGTACCGACGATCTCGTCAAAGC	ATGGTAGGTCTCAGCGCTTGCGAGTGGGGTGTCCTGTTC	BsaI	pPR-IBA1	C-terminal Strep-tag
**BPSS1525**	BPSS1525-79	ATGGTAGGTCTCAAATGGGCGACGCGAAACAGGCGATC	ATGGTAGGTCTCAGCGCTCGCGCCGTCCGCCGCGTTC	BsaI	pPR-IBA1	C-terminal Strep-tag
**BPSS1532**	BPSS1532-344	ATGGTAGGTCTCAAATGTCATCGGGAGTGCAGGGCGG	ATGGTAGGTCTCAGCGCTCTTGCCGATGCAGCCCATCGT	BsaI	pPR-IBA1	C-terminal Strep-tag
**BPSS1532**	BPSS1532-344	ATGGTAGGTCTCAAATGTCATCGGGAGTGCAGGGCGG	ATGGTAGGTCTCAGCGCTCTTGCCGATGCAGCCCATCGT	BsaI	pASK-IBA33plus	C-terminal His-tag
**BPSS1722**	BPSS1722	ATGGTAGGTCTCAAATGGCTAAGCCCGCAAAGCGCGTT	ATGGTAGGTCTCAGCGCTCTTCAGCAGATGGGCGACGCC	BsaI	pPR-IBA1	C-terminal Strep-tag
**BPSS2141**	BPSS2141	ATGGTAGGTCTCAAATGGTCACCGTTGCCTCGAATGTCA	ATGGTAGGTCTCAGCGCTGTGCTTGATCAGGTACATGTCCT	BsaI	pPR-IBA1	C-terminal Strep-tag
