# Supplementary material for: Rapid and Sensitive Multiplex Detection of Burkholderia pseudomallei-Specific Antibodies in Melioidosis Patients Based on a Protein Microarray Approach
Source: PLoS Negl Trop Dis. 2016 Jul 18;10(7):e0004847. doi: 10.1371/journal.pntd.0004847 (PMC4948818; doi:10.1371/journal.pntd.0004847)
Supplement: S3 Table — (PDF) [file pntd.0004847.s013.pdf]

Species obtained from blood culture	number
Gram-negative bacteria	
Bacteroides vulgatus	1
Escherichia coli	12
Haemophilus influenzae	2
Moraxella osloensis	1
Pseudomonas aeruginosa	4
Sphingomonas paucimobilis	1
Stenotrophomonas maltophilia	3
Raoultella (Klebsiella) ornithinolytica	2
Gram-positive bacteria	
Propionibacterium acnes	1
Staphylococcus aureus	8
Staphylococcus capitis	2
Staphylococcus cohnii	1
Staphylococcus epidermidis	2
Staphylococcus haemolyticus	2
Staphylococcus hominis	1
Streptococcus agalactiae	1
Streptococcus mitis/oralis	2
Streptococcus pneumoniae	4
Enterococcus faecalis	2
Enterococcus faecium	5
Fungus	
Candida albicans	3
total	60
