# Supplementary material for: Rapid and Sensitive Multiplex Detection of Burkholderia pseudomallei-Specific Antibodies in Melioidosis Patients Based on a Protein Microarray Approach
Source: PLoS Negl Trop Dis. 2016 Jul 18;10(7):e0004847. doi: 10.1371/journal.pntd.0004847 (PMC4948818; doi:10.1371/journal.pntd.0004847)
Supplement: S1 File — Columns represent the corresponding comparison from S5 Fig and rows show the spotted B. pseudomallei antigens. All blue stained boxes mark significantly recognised B. pseudomallei antigens in the respective comparison. Statistical analysis was performed using the Fisher's exact test. p values smaller than or equal 0.01 were assumed to differ significantly between the respective sera group. Shown are p values for every antigen spotted with a concentration of 0.45 mg/ml. (PPTX) [file pntd.0004847.s014.pptx]

## Slide 1
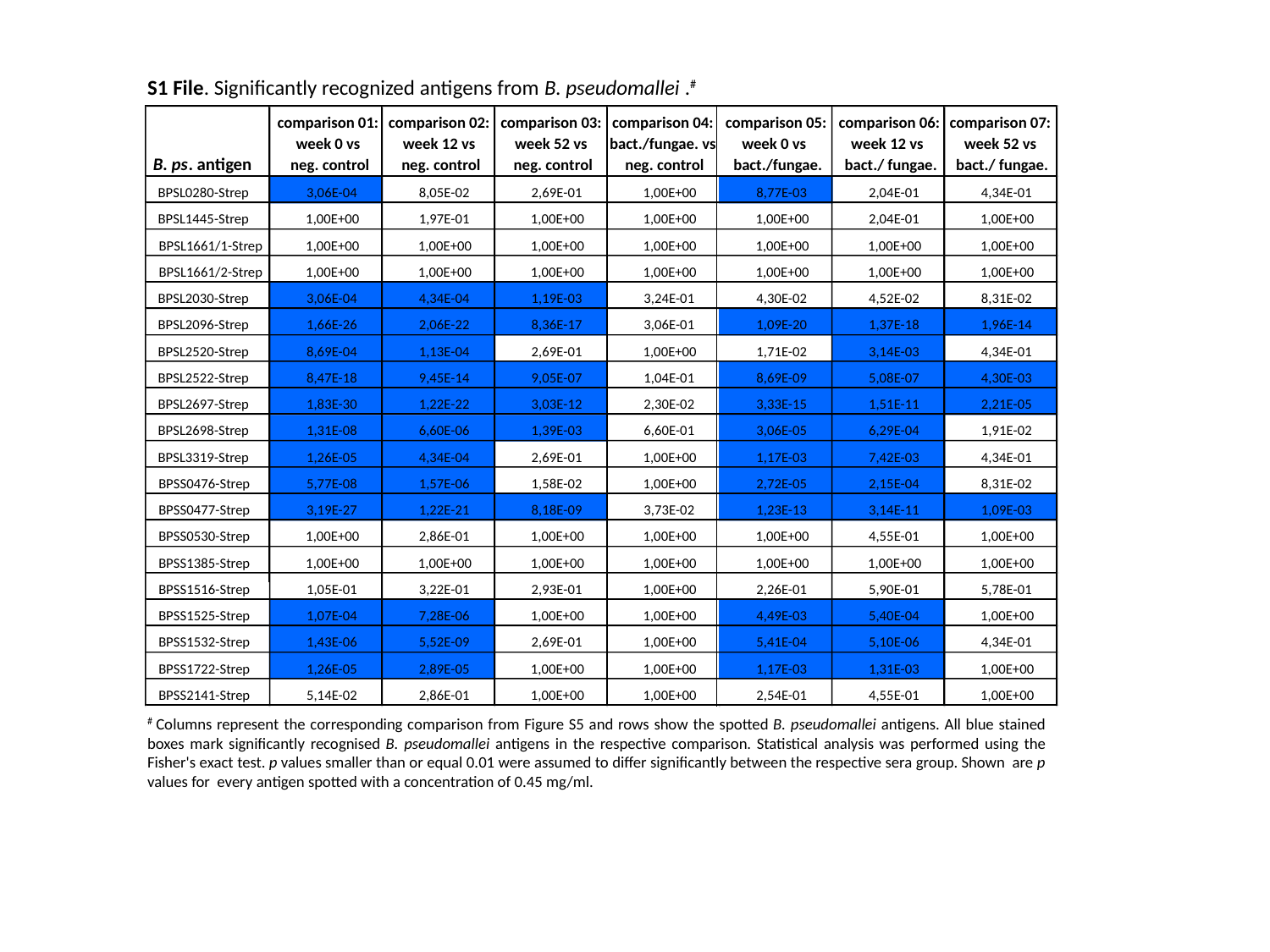

S1 File. Significantly recognized antigens from B. pseudomallei .#
comparison 01:
comparison 02:
comparison 03:
comparison 04:
comparison 05:
comparison 06:
comparison 07:
week 0 vs
week 12 vs
week 52 vs
bact./fungae. vs
week 0 vs
week 12 vs
week 52 vs
B. ps
. antigen
neg. control
neg. control
neg. control
neg. control
bact./fungae.
bact./ fungae.
bact./ fungae.
BPSL0280-Strep
3,06E-04
8,05E-02
2,69E-01
1,00E+00
8,77E-03
2,04E-01
4,34E-01
BPSL1445-Strep
1,00E+00
1,97E-01
1,00E+00
1,00E+00
1,00E+00
2,04E-01
1,00E+00
BPSL1661/1-Strep
1,00E+00
1,00E+00
1,00E+00
1,00E+00
1,00E+00
1,00E+00
1,00E+00
BPSL1661/2-Strep
1,00E+00
1,00E+00
1,00E+00
1,00E+00
1,00E+00
1,00E+00
1,00E+00
BPSL2030-Strep
3,06E-04
4,34E-04
1,19E-03
3,24E-01
4,30E-02
4,52E-02
8,31E-02
BPSL2096-Strep
1,66E-26
2,06E-22
8,36E-17
3,06E-01
1,09E-20
1,37E-18
1,96E-14
BPSL2520-Strep
8,69E-04
1,13E-04
2,69E-01
1,00E+00
1,71E-02
3,14E-03
4,34E-01
BPSL2522-Strep
8,47E-18
9,45E-14
9,05E-07
1,04E-01
8,69E-09
5,08E-07
4,30E-03
BPSL2697-Strep
1,83E-30
1,22E-22
3,03E-12
2,30E-02
3,33E-15
1,51E-11
2,21E-05
BPSL2698-Strep
1,31E-08
6,60E-06
1,39E-03
6,60E-01
3,06E-05
6,29E-04
1,91E-02
BPSL3319-Strep
1,26E-05
4,34E-04
2,69E-01
1,00E+00
1,17E-03
7,42E-03
4,34E-01
BPSS0476-Strep
5,77E-08
1,57E-06
1,58E-02
1,00E+00
2,72E-05
2,15E-04
8,31E-02
BPSS0477-Strep
3,19E-27
1,22E-21
8,18E-09
3,73E-02
1,23E-13
3,14E-11
1,09E-03
BPSS0530-Strep
1,00E+00
2,86E-01
1,00E+00
1,00E+00
1,00E+00
4,55E-01
1,00E+00
BPSS1385-Strep
1,00E+00
1,00E+00
1,00E+00
1,00E+00
1,00E+00
1,00E+00
1,00E+00
BPSS1516-Strep
1,05E-01
3,22E-01
2,93E-01
1,00E+00
2,26E-01
5,90E-01
5,78E-01
BPSS1525-Strep
1,07E-04
7,28E-06
1,00E+00
1,00E+00
4,49E-03
5,40E-04
1,00E+00
BPSS1532-Strep
1,43E-06
5,52E-09
2,69E-01
1,00E+00
5,41E-04
5,10E-06
4,34E-01
BPSS1722-Strep
1,26E-05
2,89E-05
1,00E+00
1,00E+00
1,17E-03
1,31E-03
1,00E+00
BPSS2141-Strep
5,14E-02
2,86E-01
1,00E+00
1,00E+00
2,54E-01
4,55E-01
1,00E+00
# Columns represent the corresponding comparison from Figure S5 and rows show the spotted B. pseudomallei antigens. All blue stained boxes mark significantly recognised B. pseudomallei antigens in the respective comparison. Statistical analysis was performed using the Fisher's exact test. p values smaller than or equal 0.01 were assumed to differ significantly between the respective sera group. Shown are p values for every antigen spotted with a concentration of 0.45 mg/ml.
