# Supplementary material for: Rapid and Sensitive Multiplex Detection of Burkholderia pseudomallei-Specific Antibodies in Melioidosis Patients Based on a Protein Microarray Approach
Source: PLoS Negl Trop Dis. 2016 Jul 18;10(7):e0004847. doi: 10.1371/journal.pntd.0004847 (PMC4948818; doi:10.1371/journal.pntd.0004847)
Supplement: S1 Flow Diagram — (PDF) [file pntd.0004847.s017.pdf]

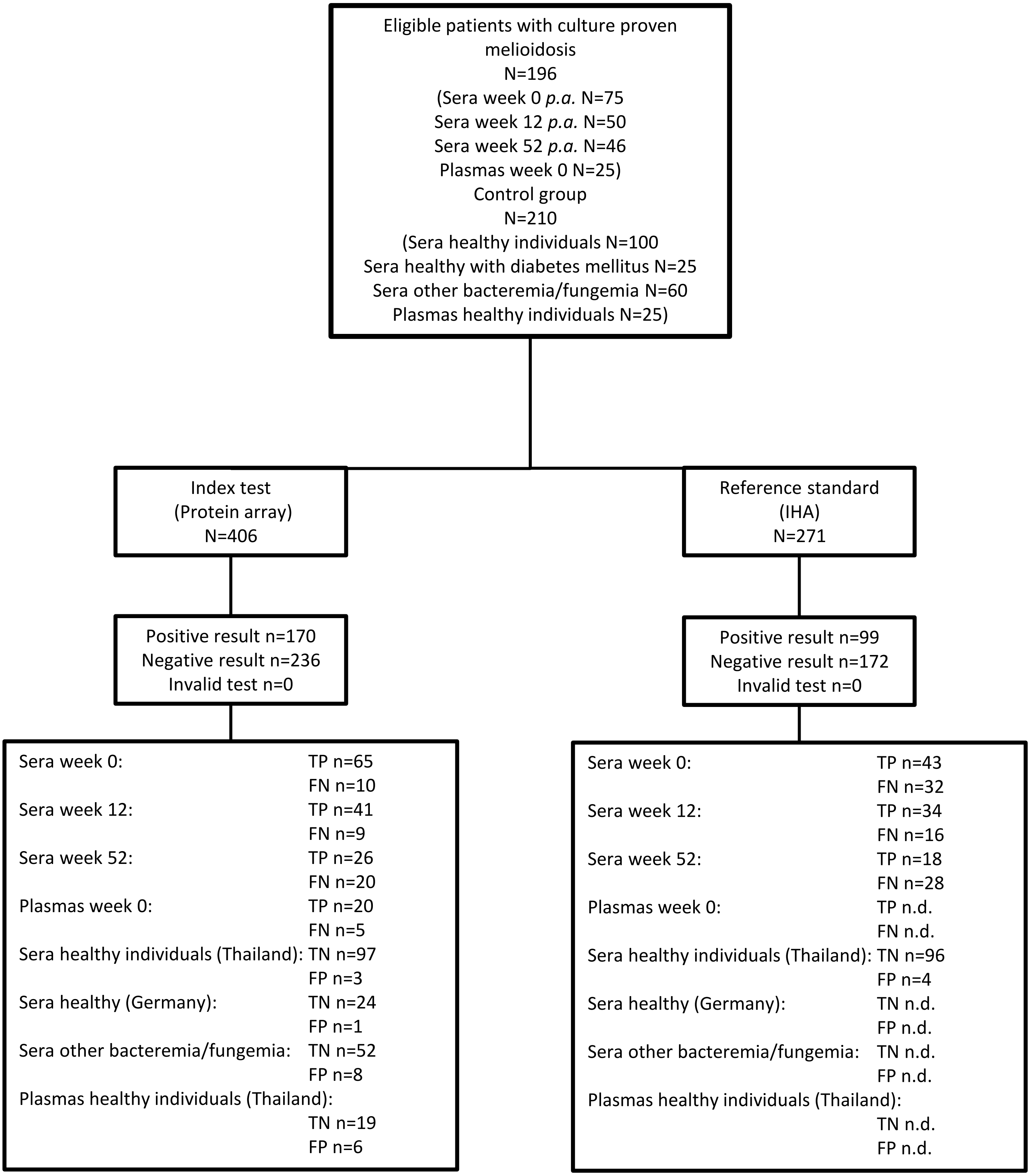

Abbr.: *p.a.* – post admission; n.d. – not determined; TP – true positive; FN – false negative; TN – true negative; FP – false positive
